# Influence of the Abiotic Elicitors Ag Salts of Aspartic Acid Derivatives, Self-Organized in Nanofibers with Monomeric and Dimeric Molecular Structures, on the Antioxidant Activity and Stevioside Content in Micropropagated *Stevia rebaudiana* Bert.

**DOI:** 10.3390/plants12203574

**Published:** 2023-10-14

**Authors:** Mariana Sichanova, Maria Geneva, Maria Petrova, Kamelia Miladinova-Georgieva, Elisaveta Kirova, Trendafil Nedev, Daniela Tsekova, Viktoria Ivanova, Antoaneta Trendafilova

**Affiliations:** 1 Institute of Plant Physiology and Genetics, Bulgarian Academy of Sciences, Acad. G. Bonchev Street, Bldg. 21, 1113 Sofia, Bulgaria; m.sichanova@abv.bg (M.S.); marry_petrova@yahoo.com (M.P.); kameca@abv.bg (K.M.-G.); elisab@abv.bg (E.K.); tanedev@abv.bg (T.N.); 2 Department of Organic Chemistry, University of Chemical Technology and Metallurgy, 8“St. Kl. Ohridski” Blvd, 1756 Sofia, Bulgaria; d_tsekova@uctm.edu; 3 Institute of Organic Chemistry with Centre of Phytochemistry, Bulgarian Academy of Sciences, Acad. G. Bonchev Street, Bldg. 9, 1113 Sofia, Bulgaria; viktoria.genova@orgchm.bas.bg (V.I.); antoaneta.trendafilova@orgchm.bas.bg (A.T.)

**Keywords:** *Stevia rebaudiana* Bert., antioxidant activity, in vitro propagation, steviol glycosides, monocaffeoylquinic acids (CQA), dicaffeoylquinic acids (DCQA)

## Abstract

The use of nanomaterials in biotechnology for the in vitro propagation of medical plants and the accumulation of certain biologically active metabolites is becoming an efficient strategy. This study aimed to evaluate the influence of the concentration (0, 1, 10, 50, and 100 mg L^−1^) of two types of nanofibers on the growth characteristics, the antioxidant status, and the production of steviol glycosides in micropropagated *Stevia rebaudiana* Bert. plantlets. The nanofibers were synthesized by aspartic acid derivatives (L-Asp) Ag salts self-organized into nanofibers with two different molecular structures: monomeric, containing one residue of L-Asp with one hydrophilic head which bonds one Ag ion (NF1-Ag salt); and dimeric, containing two residues of L-Asp with two hydrophilic heads which bond two Ag ions (NF2-Ag salt). An increase in the shoots from the explants’ number and length, biomass accumulation, and micropropagation rate was achieved in the plants treated with the NF1-Ag salt in concentrations from 1 to 50 mg L^−1^ after 30 days of in vitro proliferation compared to the NF2-Ag salt. In contrast, the plants grown on MS media supplemented with NF2-Ag salt exhibited an increase in the level of stevioside, rebaudioside A, and mono- (CQA) and dicaffeoylquinic (DCQA) acids as compared to the NF1-Ag salt.

## 1. Introduction

*Stevia rebaudiana* Bertoni (sweet leaf, sweet herb of Paraguay, honey leaf, or candy leaf) is a native plant from South America and is one of the most preferred natural sweeteners in recent years. The plant’s sweetness is due mainly to steviol glycosides, which are 250–300 times sweeter than table sugar. The stevioside and rebaudioside A (the two main diterpenoid steviol glycosides), are present in the plant’s leaves. Since the body does not metabolize the glycosides in stevia, the leaves have been employed as a low-calorie sweetener [1]. The other compounds extracted from stevia leaves are flavonoids, alkaloids, chlorophylls, xanthophylls, hydroxycinnamic acids (caffeic, chlorogenic, etc.), oligosaccharides, free sugars, amino acids, and lipids [2,3]. The plant exhibits several biological activities including anti-hypertensive, anti-obesity, anti-diabetic, antioxidant, anti-cancer, anti-inflammatory, and antimicrobial effects, and improvement of kidney function [4]. 

Due to its highly beneficial effect on human health, scientists’ efforts focus on cultivating plants and improving their productivity. The role of nanoparticles as inducers of abiotic stress in plants has been studied for years [5]. Currently, nanoparticles are increasingly used in plant biotechnology for callus induction improvement, organogenesis, and somatic embryogenesis, as well as for accelerating growth and enhancing secondary metabolite accumulation [6]. Nanoparticles, which are used in in vitro plant cell and tissue studies are applied at different sizes, shapes, ion concentrations, and types of surface coating [7]. They interact with plants causing various morphological and physiological changes depending on their properties [8]. Nanoparticles can act as an elicitor or nutrient source, thus enhancing secondary metabolite production in micro-propagated plants [9]. The Ag nanoparticles (AgNP) used in the in vitro culture of *S. rebaudiana* were found to have a positive effect on shoot proliferation and length, photosynthetic pigment synthesis, nutrient accumulation, antioxidant metabolism, ROS generation, and steviol glycosides levels [10,11]. AgNPs influence the studied parameters in a concentration-dependent manner. The plant’s response to a variety of chemical and physical agents is dose-dependent, with low-dose stimulation and high-dose inhibition observed, and this phenomenon is called hormesis [12]. Spinoso-Castillo et al. [13] evaluated the hormetic effect of silver nanoparticles (Argovit™) on the in vitro regeneration of *Vanilla planifolia* Jacks. Ex-Andrews. AgNPs have been added to the Murashige and Skoog (MS) liquid medium at five different concentrations (0, 25, 50, 100, and 200 mg L^−1^). Growth stimulation was achieved with 25 and 50 mg L^−1^ of AgNPs, while 100 and 200 mg L^−1^ concentrations caused significant inhibition [13]. The concentration range of 1, 10, 50, and 100 mg L^−1^ was selected to allow for the establishment of the hormetic effect of the tested nanofibers. The positive effect of the AgNPs on micro-propagation was reported in many other plant species [14,15,16]. The reactive oxygen species (ROS) produced after the application of AgNPs into cells leads to the induction of cellular signaling pathways, essentially the mitogen-activated protein (MAP) kinase pathway, which promotes the growth and development of plants [5,17]. Numerous studies showed the beneficial effect of silver nitrate (AgNO_3_) on plant regeneration, shoot multiplication, and root formation [18,19,20]. In addition, AgNO_3_ can be used as an elicitor for the biosynthesis of secondary metabolites in plants. Due to its water solubility and lack of phytotoxicity at proper doses, AgNO_3_ has been used in tissue culture research to inhibit the action of ethylene [21]. The silver nitrate inhibits ethylene action through the Ag^2+^ ions, by reducing the receptor capacity to bind ethylene [22,23]. This process increases the production of secondary metabolites, plant regeneration, and the rate of micropropagation. The morphogenesis and growth of plant tissue under in vitro conditions depend on the composition of the nutrient medium: macro- and micro-salts, vitamins, carbohydrates, amino acids, and plant growth regulators (PGRs) [24]. Amino acids provide an essential quick nitrogen source in plants as compared with inorganic nitrogen [25]. The amino acid asparagine produced the healthiest vigorous shoots, the lowest withering percentages, and took a maximum number of days to show withering of *Rosa centifolia* shoots [26]. The growth parameters of *Phaseolus vulgaris* in vitro plants increased under the asparagine and glutamine treatment effect, applied in low concentrations [27]. The low concentration of asparagine showed a slight increase in the maize embryogenic callus and the high concentration suppresses the callus growth [28]. However, there is evidence of an improvement in the somatic embryogenesis of *Triticum aestivum* L. in the presence of asparagine in a nutrient medium [29]. All four studied amino acids (including asparagine) influenced the leaf chlorophyll, carotenoid, or porphyrin content, sugar biosynthesis and metabolism, and proline accumulation in both the leaves and roots of the in vitro cultured cherry rootstocks [30]. Alpha-amino acids and their derivatives are very promising types of additives acting either as active compounds or as carriers of other active compounds. There are many reports about amphiphilic molecules, alpha-amino acids derivatives that possess biological activity or take part in supramolecular structures, being loaded with other substances [31,32].

Although many researchers have studied the antioxidant activity and phytochemical composition of *S. rebaudiana* Bert., there is no information on the caused changes in *S. rebaudiana* in vitro propagated with aspartic acid derivatives. We suggest the nanostructured silver salts of aspartic acid (L-Asp) derivatives’ addition to the MS nutrient medium of *S. rebaudiana* Bert. Here, we present the obtainment of silver salts of aspartic acid derivatives with two different molecular structures: monomeric, containing one residue of L-Asp with one hydrophilic head which bonds one Ag ion (NF1 Ag salt); and gemini, containing two residues of L-Asp with two hydrophilic heads which bind two Ag ions (NF2 Ag salt). This study’s objective was to determine the stevioside composition and antioxidant activity of *S. rebaudiana* in vitro plantlets treated with both silver salts self-organized into spherical nanoparticles ordered in a line, like a fiber, as sources of silver ions, using different concentrations.

## 2. Results

### 2.1. Morphological Evaluation

Addition to the cultivation MS medium of NF1-Ag salt (nanofibers from silver salts of aspartic acid derivatives with monomeric molecular structure) in concentrations from 1 to 50 mg L^−1^ exhibited a beneficial effect on the growth of *S. rebaudiana* plantlets—by increasing the weight and number of shoots compared to the control plantlets (Table 1) (Figure S1). A concentration of 100 mg L^−1^ of nanofiber significantly inhibited the growth, with reduced height, weight, and number of shoots.

Adding NF2-Ag salt to the MS medium led to shoot DW and height reduction, compared with the nontreated control plants. Meanwhile, the micropropagation rate increases, due to the increased number of newly produced shoots and internodes. Changes in the concentration from 1 to 50 mg L^−1^ of NF2-Ag salt added to the MS cultivation medium, had no significant effect on the *S. rebaudiana* plantlets’ biomass accumulation and shoot number per explant (Table 2) (Figure S2). Only a decrease in the shoot height was recorded. In addition, a lack of significant changes in the shoot number and a decrease in the internode number leads to a reduction in the micropropagation rate, which is determined by the number of newly produced shoots and nodes.

The highest micropropagation rate, MR, was recorded in the *S. rebaudiana* plantlets grown in the MS medium with the addition of 50 mg L^−1^ NF1-Ag-salt (15.04) and 1 mg L^−1^ NF2-Ag-salt (5.95) (Table 1 and Table 2). When evaluating the effect of the nanofibers on the in vitro propagation of stevia, the initiation of root formation was noted, which is not recorded in the cultivation of plants in the MS medium free of NFs. The root formation reached the highest percentage (8%) at 1 mg L^−1^ NF1-Ag-salt, and then at 10 mg L^−1^ it decreased to 5.13%, maintaining the same values at 100 mg L^−1^ NF1-Ag-salt. A high root formation rate was registered at all four tested concentrations of NF2-Ag-salt, with the highest value at 10 mg L^−1^ NF2-Ag-salt (62.5%).

### 2.2. Antioxidant Enzyme Activity

**Figure 1:**
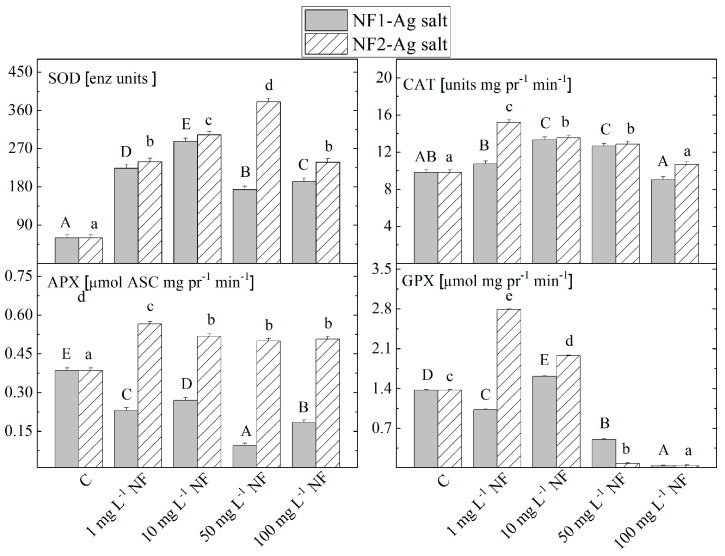
The activity of antioxidant enzymes superoxide dismutase (SOD), catalase (CAT), ascorbate peroxidase (APX), and guaiacol peroxidase (GPX) in *S. rebaudiana* plantlets in vitro propagated on MS media I, and on MS media supplemented with various concentrations (1, 10, 50, 100 mg L^−1^) of nanofibers synthesized by the derivatives of the L-aspartic acid with a monomeric (NF1-Ag salt) or dimeric molecular structure (NF2-Ag salt) carrier of silver ions. Values are means ± SE, *n* = 20; different letters indicate significant differences assessed by the Fisher LSD test (*p* ≤ 0.05) after performing ANOVA one-way analysis. We used the letter “a” or “A” for the lowest data value and ascending to the next letters for higher data value. The statistical analysis of NF1-Ag salt (uppercase) and NF2-Ag salt (lowercase) was performed separately.

According to the untreated control plants, the activity of SOD, the first enzyme that eliminates superoxide anions by transforming them into H_2_O_2_ and O_2_, increases with the addition of nanofibers from 1 to 10 mg L^−1^ for NF1-Ag salt and up to 50 mg L^−1^ for NF2-Ag salt in the nutrient medium (Figure 1). The subsequent increase in the nanofibers’ concentration led to a reduction in the SOD activity.

The activity level of the enzyme that converts H_2_O_2_ into H_2_O and O_2_, CAT is raised by an increase in the concentrations of NF1-Ag salt from 1 mg L^−1^ to 10 mg L^−1^, and with subsequent nanofiber concentration increasing, it decreased. When treated with NF2-Ag salt, an inversely proportional relationship was recorded between the organic NF concentration and the enzyme activity in the stevia plantlets. The APX activity reduction was registered in stevia plantlets cultivated on 1 to 100 mg L^−1^ NF1-Ag salt, while when treated with NF2-Ag salt at all studied concentrations, the activity of APX increased and did not change significantly with varying concentrations. The GPX activity decreased significantly when the MS nutrient medium was supplied with NF1-Ag salt. Only at a concentration of 10 mg L^−1^, the level was higher. An increase in the GPX activity is recorded only at treatment with 1 and 10 mg L^−1^ NF2-Ag salt. Then, with an increase in concentration, the activity decreases.

### 2.3. Non-Enzymatic Antioxidant Activity

**Figure 2:**
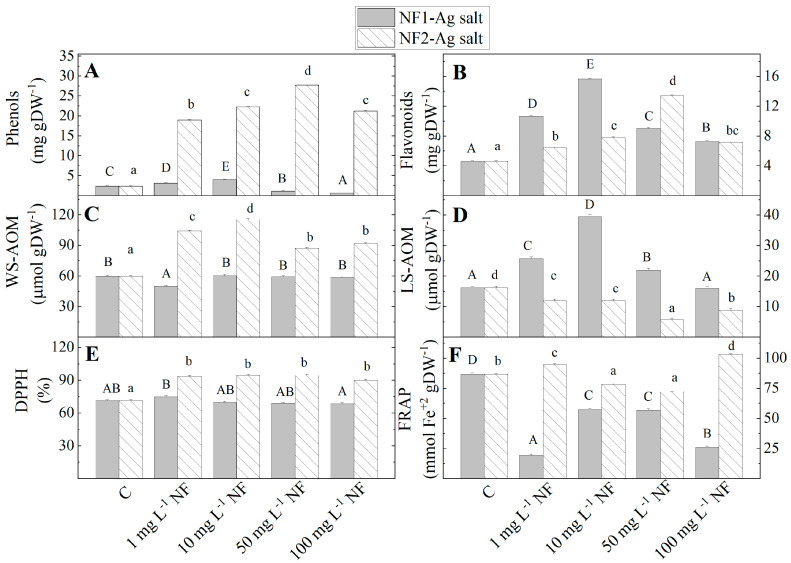
The content of metabolites with antioxidant power (total phenolic compounds (**A**) and flavonoids (**B**), WS-AOM (**C**) and LS-AOM (**D**)) and antioxidant potential (DPPH (**E**), FRAP (**F**)) in *S. rebaudiana* plantlets in vitro propagated on MS media, and on MS media supplemented with various concentrations (1, 10, 50, 100 mg L^−1^) of nanofibers synthesized by the derivatives of the L-aspartic acid with a monomeric (NF1-Ag salt) or dimeric molecular structure (NF2-Ag salt) carrier of silver ions. Values are means ± SE, *n* = 20; different letters indicate significant differences assessed by the Fisher LSD test (*p* ≤ 0.05) after performing ANOVA one-way analysis. We used the letter “a” or “A” for the lowest data value and ascending to the next letters for higher-data value. The statistical analysis of NF1-Ag salt (uppercase) and NF2-Ag salt (lowercase) was performed separately.

After four weeks of culture on MS media with 1 and 50 mg L^−1^ NF1-Ag salt, the level of total phenolic content increased in the stevia plantlets, compared to the control plants (C), while the higher concentration (100 mg L^−1^) of NF1-Ag salt led to a decrease (Figure 2). The treatment with NF2-Ag salt leads to the phenol content decrease at all tested concentrations against the controls. The amount of the flavonoids increased with an increase in the concentrations of NF1-Ag salt from 1 to 10 mg L^−1^ and NF2-Ag salt from 1 to 50 mg L^−1^, after which it began to reduce but remained higher than the controls (C). A significant increase in the content of water-soluble metabolites with antioxidant potential was statistically recorded upon the addition of NF2-Ag salt to the MS medium at the four tested concentrations. However, the treatment with NF1-Ag salt did not lead to important changes in the water-soluble metabolites content.

Comparing the total antioxidant potential measured by both methods, by the radical scavenging capacity (DPPH method) and ferri reducing antioxidant power (FRAP method), differences in their values were found depending on the type of nanofibers. The addition of the NF1-Ag salt does not affect the radical scavenging capacity, while the FRAP was the lowest at 1 mg L^−1^, then it increased at 10 and 50 mg L^−1^. The MS nutrient medium enrichment with NF2-Ag salt increased the radical scavenging capacity compared to the control, where its levels remained the same at each of the four studied concentrations.

### 2.4. Oxidative Stress Markers

**Figure 3:**
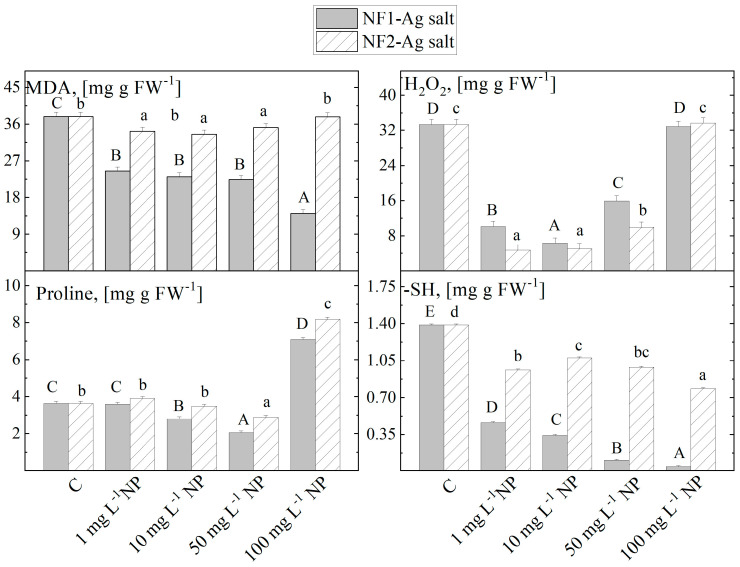
The levels of stress markers (MDA, H_2_O_2_, proline, and -SH groups) in the *S. rebaudiana* plantlets in vitro propagated on MS media, and on MS media supplemented with various concentrations (1, 10, 50, 100 mg L^−1^) of nanofibers synthesized by the derivatives of the L-aspartic acid with a monomeric (NF1-Ag salt) or dimeric molecular structure (NF2-Ag salt) carrier of silver ions. Values are means ± SE, *n* = 20; different letters indicate significant differences assessed by the Fisher LSD test (*p* ≤ 0.05) after performing ANOVA one-way analysis. We used the letter “a” or “A” for the lowest data value and ascending to the next letters for higher-data value. The statistical analysis of NF1-Ag salt (uppercase) and NF2-Ag salt (lowercase) was performed separately.

The added NF1-Ag salt to the nutrient medium in concentrations from 1 to 50 mg L^−1^ significantly reduced the content of the MDA in *S. rebaudiana* plantlets compared to the level in the control plants grown in NFs-free media, while the highest concentration of NF1-Ag salt at 100 mg L^−1^ lead to an additional decrease (Figure 3). The MS nutrient medium enrichment with NF2-Ag salt at increasing concentrations does not considerably affect the MDA content.

The H_2_O_2_ content in the *S. rebaudiana* plants treated with the tested two molecular structure type organic nanofibers (NF1-Ag salt and NF2-Ag salt) at concentrations 1 to 50 mg L^−1^ was reduced. Only at the concentration of 100 mg L^−1^ a higher value was reached, comparable to those observed in the control. At 1 mg L^−1^ NF1-Ag salt in the MS nutrient medium, the concentration of the other stress marker proline is as much as the control untreated plants. The increase in the nanoparticles to 50 mg L^−1^ NF1-Ag salt decreased the quantity in the *S. rebaudiana* plantlets, while 100 mg L^−1^ NF1-Ag salt increased the proline level. The treatment with the two types of nanofibers with monomeric and dimeric molecular structures led to a gradual decrease in the values recorded for the sulfhydryl groups, with an increase in the nanoparticle concentrations, and this decrease is much more pronounced in the NF1-Ag salt variants.

### 2.5. Content of Diterpenoid Steviol Glycosides (Stevioside and Rebaudioside A) and Mono and Di Chlorogenic Acids

**Figure 4:**
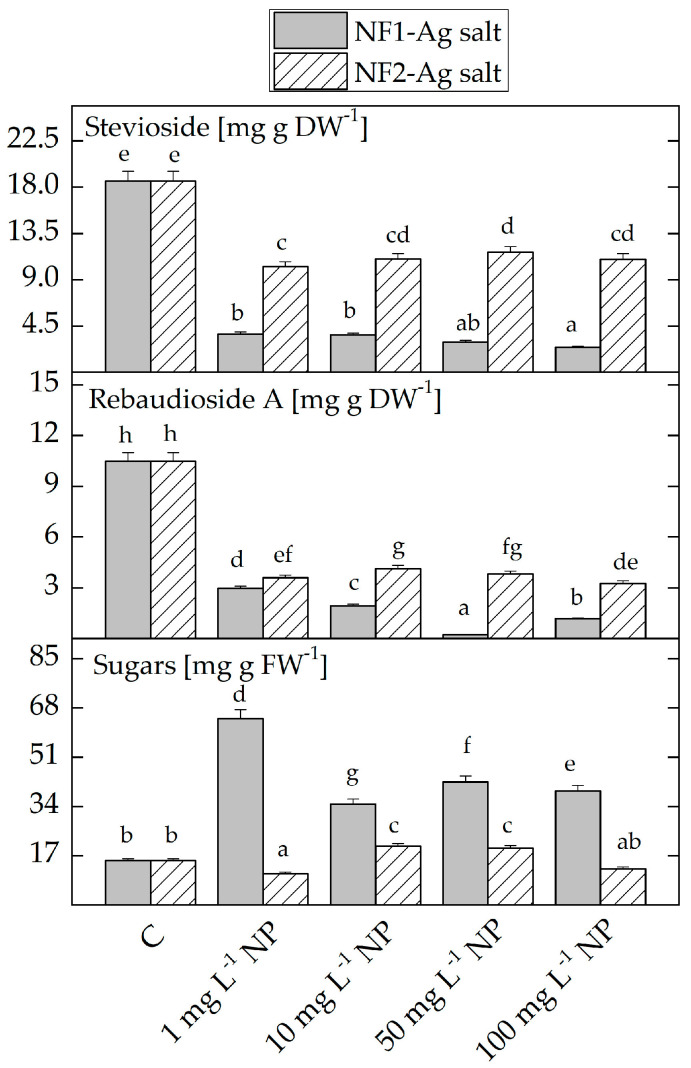
Stevioside, rebaudioside A, and sugar content in the *S. rebaudiana* plantlets in vitro propagated on MS media free of NP, and on MS media supplemented with various concentrations (1, 10, 50, 100 mg L^−1^) of nanofibers synthesized by the derivatives of the L-aspartic acid with a monomeric (NF1-Ag salt) or dimeric molecular structure (NF2-Ag salt) carrier of silver ions. Values are means ± SE, *n* = 6; different letters indicate significant differences assessed by the Fisher LSD test (*p* ≤ 0.05) after performing ANOVA one-way analysis. We use the letter “a” for the lowest data value and ascend to the next letters for higher data value.

Reducing the content of stevioside and rebaudioside A was registered in the *S. rebaudiana* plantlets grown with NF1-Ag salt and NF2-Ag salt, compared to the untreated control plants, while the total soluble sugar content increased (Figure 4). When plantlets were treated with NF2-Ag salt, the stevioside and rebaudioside A levels declined less compared to the NF1-Ag salt. A more significant decrease in the stevioside and rebaudioside A levels correlates with greater increases in the concentration of the total sugars. The *S. rebaudiana* plantlets treated with 1 mg L^−1^ NF1-Ag salt had the highest measured total sugar content.

**Figure 5:**
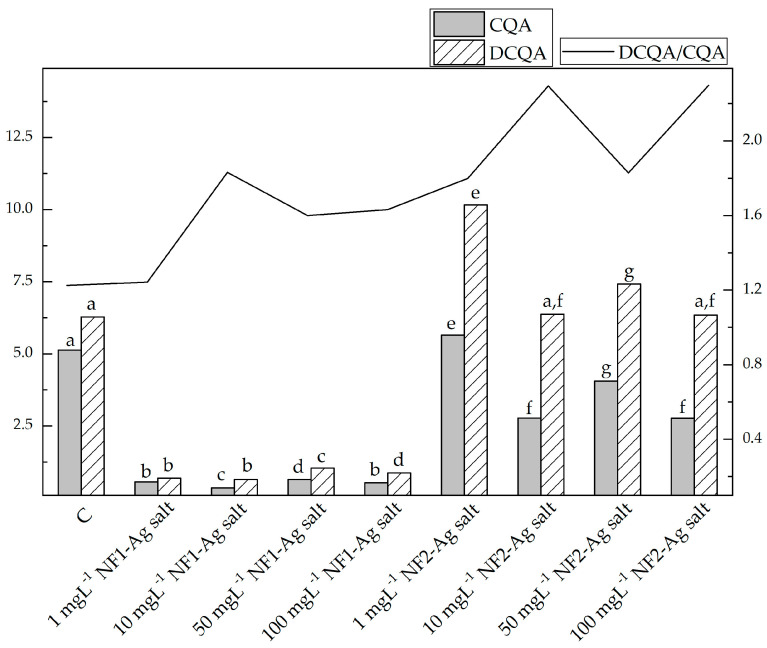
Content of total mono- (CQA) and dicaffeoylquinic (DCQA) acids (mg/g DW) and CQA/D CQA ratio in the *S. rebaudiana* plantlets in vitro propagated on MS media free of NP, and on MS media supplemented with various concentrations (1, 10, 50, 100 mg L^−1^) of nanofibers synthesized by the derivatives of the L-aspartic acid with a monomeric (NF1-Ag salt) or dimeric molecular structure (NF2-Ag salt) carrier of silver ions. Values are means ± SE, *n* = 6; different letters indicate significant differences assessed by the Fisher LSD test (*p* ≤ 0.05) after performing ANOVA one-way analysis. We use the letter “a” for the lowest data value and ascend to the next letters for higher data value.

The results from the quantitative determination of mono- (CQA) and dicaffeoylquinic (DCQA) acids and quercetin-3-O-rhamnoside (Qu-3-Rha) (mg/g DM) in the *S. rebaudiana* plantlets are represented in Table 3 and Figure 5. As can be seen, chlorogenic acid (5-CQA) was the major component among the monocaffeoyl esters of quinic acid in all samples and was detected in amounts from 0.26 to 4.80 mg g^=1^ DM. The content of neochlorogenic (3-CQA) and cryptochlorogenic (4-CQA) acid was significantly lower and varied from 0.04 to 0.35 mg g^=1^ DM and from 0.03 to 0.58 mg g^=1^ DM, respectively. Among dicaffeoylquinic acids, 3,5-DCQA was the principal component (0.32–6.84 mg g^=1^ DM) followed by 4,5-DCQA (0.17–2.76 mg g^−1^ DM) and 3,4-DCQA (0.07–0.57 mg g^−1^ DM). The total amount of DCQA was higher than that of CQA in all samples and the DCQA/CQA ratio varied from 1.22 (C, MS free of NF) to 2.3 (NF2-Ag salt at 10 mg L^−1^ and 100 mg L^−1^) (Figure 4). The lowest CQA and DCQA content was found in the *S. rebaudiana* plantlets in vitro propagated on MS media supplemented with various concentrations (1, 10, 50, 100 mg L^−1^) of the nanofibers synthesized by the derivatives of the L-aspartic acid with a monomeric structure carrier of silver ions (NF1-Ag salt). The content of the CQA and DCQA in the samples treated with NF2-Ag salt was 2.8–5.70 and 6.3–10.2 times higher than that in the samples treated with NF1-Ag salt. The plantlets treated with 1 mg L^−1^ NF2-Ag salt were the richest in CQA and DCQA and their amount exceeded that in the plantlets propagated on MS media free of NP (C) with the exception of 3-CQA.

The content of quercetin 3-rhamnosdie (Qu 3-Rha) was relatively low and varied from 0.08 to 0.61 mg g^−1^ DM. The plantlets treated with NF2-Ag salt contained similar or higher amounts of Qu 3-Rha compared to those grown on NP-free MS Imedia (C).

**Figure 6:**
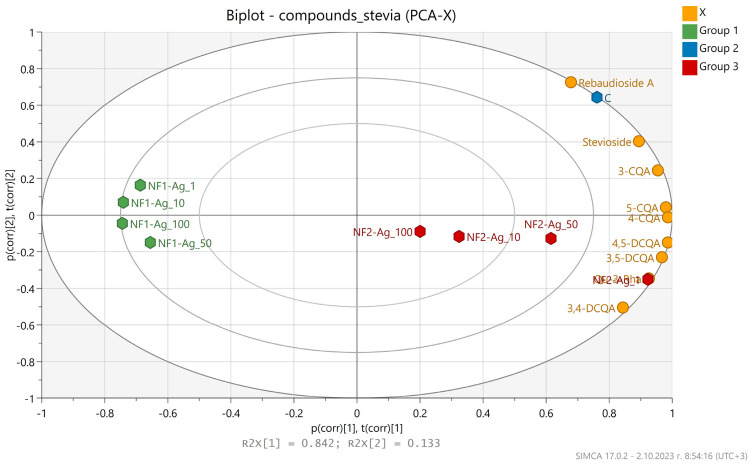
Biplot from PCA performed on the mean content of the individual compounds in different extracts of *Stevia rebaudiana* plantlets in vitro propagated on MS media free of NP, and on MS media supplemented with nanofibers synthesized by the derivatives of the L-aspartic acid with a monomeric (NF1-Ag salt) or dimeric molecular structure (NF2-Ag salt) carrier of silver ions.

A principal component analysis (PCA) was used to establish the differences in the chemical compositions of the *S. rebaudiana* plantlets in vitro propagated on MS media free of NP, and MS media supplemented with various concentrations (1, 10, 50, 100 mg L^−1^) of nanofibers synthesized by the derivatives of the L-aspartic acid with a monomeric (NF1-Ag salt) or dimeric molecular structure (NF2-Ag salt) carrier of silver ions. The PCA performed on the content of individual compounds—mono- and dicaffeoylquinic acids, quercetin 3-O-rhamnoside, stevioside, and rebaudioside A—in the extracts showed that the first principal axes PC1 and PC2 accounted for 84.2 and 13.3% of the total variation, respectively (Figure 6). The samples were grouped into three clusters on the PCA score plot. The PC1 axis of the PCA biplot showed a separation pattern between the NF1-Ag salts and the rest due to the lowest phenolic compounds, stevioside and rebaudioside A content. The PC2 axis showed two separation patterns corresponding to C (MS media free of NP) and NF2-Ag samples. Sample C possessed the highest amount of stevioside and rebaudioside A, while phenolics dominated the NF2-Ag series samples. The 1 mg L^−1^ NF2-Ag salt sample occupied the most isolated place due to the highest content of Qu-3-Rha, 3,5-, 3,4-, and 4,5-DCQA.

A correlation analysis of the phenolic content (TPC, flavonoids, and individual compounds—quantified through HPLC-DAD) and the antioxidant activities (DPPH and FRAP) was performed with a Pearson’s correlation test (Table 4). There was a strong correlation between the TPC in the studied extracts and the antioxidant capacity assessed with the DPPH test (r = 0.971). Among the individual compounds, quercetin 3-O-rhamnoside (r = 0.885), 3,4-DCQA (r = 0.847), 3,5-DCQA (r = 0.827), and 4,5-DCQA (r = 0.808) showed the highest correlation with DPPH radical scavenging activity. The antioxidant capacity assessed with the FRAP test showed a significant correlation with 4,5-DCQA (r = 0.828), 3,5-DCQA (r = 0.823), and 3,4-DCQA (r = 0.791). All other compounds also exhibited good correlations with the DPPH and FRAP tests. The observed differences could be attributed to the different abilities of the antioxidant compounds in the extracts to quench DPPH free radicals in in vitro systems or to reduce the complex of ferric ions (Fe^3+^)-ligand to the intensely blue ferrous complex (Fe^2+^).

## 3. Discussion

For the organic nanofibers, the addition of carriers of silver ions (NF1-Ag salt, NF2-Ag salt) to MS cultural media positively affects the shoot multiplication, shoot length, and root formation in *S. rebaudiana*. Castro-González et al. [10] have observed significant differences in the parameters characterizing growth, evaluating the effect of AgNPs in different concentrations on the in vitro propagation of stevia. The lower concentrations of AgNPs added to the nutrient medium are the most favorable for the new shoots, with the highest length formation. At the highest concentration (200 mg L^−1^) of AgNPs in the MS medium, they have registered the smallest number of new shoots with the lowest height.

L-aspartic acid derivatives were used for the investigated nanofibers’ organic component synthesis. It can be assumed that the initiation of root formation in the MS medium supplemented with the peptidomimetics formed by the L-aspartic acid derivatives, is also due to the presence of an amino acid. The growth of *Arabidopsis thaliana* plants is significantly inhibited when valine is used as a source of nitrogen [33]. High concentrations of phenylalanine, valine, or proline at 10 g L^−1^ inhibited peach seedling growth (reduced height and shortened internode length), and positively influenced the root growth, with the most significant effect of valine [34]. Moreover, treatment with valine also increases the soluble sugars in the leaves and stems. It can be assumed that the initiation of root formation and the increased sugar content of the *S. rebaudiana* plantlets, grown with the addition of NF1-Ag salt and NF2-Ag salt to the MS cultural medium, are due to L-aspartic acid in the organic part of the studied nanofibers.

The treatment with amino acids during the in vitro propagation of plants affects the growth parameters differently. For example, in in vitro propagated *Vitis labrusca* cv. Bordô, the arginine favors multiplication, while in *Vitis vinifera* cv. Chardonnay, the highest number of new shoots has been reported when glycine is added to the nutrient medium [35]. Regarding the degree of root formation, the best effect was observed with alanine in *Vitis labrusca* cv. Bordô, and glycine and arginine in *Vitis vinifera* cv. Chardonnay. The beneficial or inhibitory effect of amino acids on the growth of plant tissue cultures depends on the type (chemical nature) and the amount of amino acids. Yıldırım et al. [36], have studied the influence of different amino acids (valine, tryptophan, alanine, leucine, and methionine) with varying concentrations on the parameters characterizing shoot proliferation (number and length of plantlets, internode length) of lentisk (*Pistacia lentiscus* L.). The authors reported the highest number of shoots at 100 mg L^−1^ valine added to the MS nutrient medium compared to the other investigated amino acids. The addition of alanine at a lower concentration of 25 mg L^−1^ has reduced the number of shoots compared to the control untreated plants, while a concentration increase has positively affected the shoot‘s formation and length. In contrast to the results obtained in the study, when the medicinal plant Stevia was treated with silver chemically attached to nanofibers consisting of an organic part (amino acid), spraying the leaves with a solution of 400 mg L^−1^ pure Ag NPs resulted in the highest fresh and dry weight of stevia shoots, while when sprayed with lower concentrations of silver nanoparticles from 80 to 200 mg L^−1^ Ag NP, the highest content of steviol glycosides was reported [11]. The authors reported that low concentrations of Ag NPs have had a beneficial effect on the stevia’s physiological, biochemical, and morphological characteristics, leading to an increase in the glutathione content and total antioxidant capacity and a decrease in the MDA levels. The different changes in the growth and antioxidant parameters of stevia plantlets in the experiments with silver carrier organic nanofibers are due to both the influence of the silver ions and the organic part (self-organizing into nanofibers/nanostructure complexes based on the physical interactions formed in low molecular weight L-aspartic acid (L-Asp) derivatives forming two different types of molecular structures: a monomeric structure that contains one asparagine acid residue (one hydrophilic head), to which one silver atom is attached, or a dimeric one that contains two asparagine acid residues, to which two silver atoms are attached). Additionally, even at the maximum tested concentration of 100 mg L^−1^ NF-1Ag salt and NF-2Ag salt, the amount of active silver in the MS nutritional medium was lower than when Ag was in the form of AgNPs.

Analyzing the influence of the addition of carriers of silver to the nutrient medium on the activity of antioxidant enzymes in organic nanofibers revealed a positive influence of the applied nanoparticles in lower concentrations on the SOD and CAT enzymes’ activity. When AgNPs were added to the nutrient medium at increasing concentrations, the SOD and CAT activity increased in the shoots and roots of *Lycopersicon esculentum* [37]. The increased activity of CAT and SOD has also been observed in the callus tissue of sugarcane (*Saccharum* sp.) propagated on a medium supplemented with 20 to 60 ppm AgNP [38]. At low concentrations of Cu_2_O and Zn nanoparticles, a significant increase in the activity of these two antioxidant enzymes has been reported in cucumber (*Cucumis sativus* L.), while an increased concentration of metal nanoparticles in the nutrient medium (over 100 mg L^−1^) significantly reduced their activity [39]. Similar results were observed in our investigation, where the SOD and CAT activities in plantlets treated with 100 mg L^−1^ NF dropped to a level nearly identical to that of the control compared to all other concentrations. For the other two enzymes (APX and GPX), we observed a lower activity in the plants grown on media supplemented with NF1-Ag salt compared to the control untreated plants. These results suggest that the enzyme complex in the form of SOD and CAT represents part of the defense mechanism against oxidative damage caused by the silver nanofiber carriers. Tripathi et al. [40], reported that silver nanoparticles (at concentrations ranging from 100 to 300 µM) significantly stimulated the activities of SOD and APX, while not affecting the other antioxidant enzymes activities in *Pisum sativum*.

In *S. rebaudiana* plantlets grown on MS media with an added nanomimetic formed by L-aspartic acid derivatives linked in a dimeric molecular structure to which two silver atoms are attached (NF2-Ag salt), high levels of antioxidant protection by the SOD and CAT enzymes were measured, as well as significantly higher APX and GPX activity compared to that which was NF1-Ag salt-triggered. In an experiment conducted to investigate the effects of silver nanoparticles (0, 25, 50, 75, and 100 mg L^−1^) in tomato plants, a significant increase in the activities of SOD, CAT, and POX in shoots and roots was also detected, but only at a concentration of 100 mg L^−1^ SOD activity was it decreased significantly [41]. The differences in the changes in the activities of the studied antioxidant enzymes in stevia plantlets under the influence of NF1-Ag salt and NF2-Ag salt can be assumed to be due to the doubled amount of silver ions in NF2-Ag salt, compared to NF1-Ag salt.

Plant cells utilize non-enzymatic compounds like phenols and flavonoids in addition to endogenous antioxidant enzymes under stress conditions to reduce the deleterious effects of reactive oxygen species. Phenolic compounds are the main constituents of antioxidants in most plant species and their antioxidant activity is mainly due to their redox properties. As a result, they can act as reducing agents in the free radicals’ neutralization and the reduction of metal ions to metal nanoparticles [42]. The analysis of the effect of the investigated nanofibers (NF-1Ag salt and NF-2Ag salt) at the four tested concentrations on the amount of metabolites with antioxidant capacity in *S. rebaudiana* plantlets showed that the amount of total phenols and flavonoids increased at the lower concentrations of 1 and 10 mg L^−1^ NF-1Ag salt, and then when the nanofiber concentrations increased, their levels decreased. Proline also exerts a positive effect on the total phenolic content only at low concentrations of 2.0 µM, and higher concentrations (3, 4, 5 µM) significantly reduce their levels in stevia callus [43]. The difference in the results when adding proline or a peptidomimetic formed from L-aspartic acid derivatives can be due to various factors. One of them could be the chemical structure of the amino acids. Aspartic acid contains an α-amino group and a carboxylic acid, while the proline has a secondary amine group, called an imine. Aspartate plays an essential role in plant resistance to abiotic stress, such as cold stress, drought stress, salt stress, or heavy metal stress [44].

Probably, plants exposed to stress use different defense systems and do not include the full potential of antioxidant enzymes. In this case, NF1-Ag salt activates the lipid-soluble antioxidants. In contrast, the NF2-Ag salt addition stimulated the water-soluble antioxidants and decreased the lipid-soluble antioxidant metabolites.

Changes in the stress marker (GSH, proline, MDA, etc.) levels were also observed upon the plant cells’ exposure to oxidative stress, in addition to activations of the mechanisms of enzyme and non-enzymatic antioxidant activity. The MDA level in plant tissue is a typical membrane damage indicator in plant cells. It is essential to consider that the formation of MDA is caused not only by non-enzymatic lipid peroxidation processes due to the presence of highly reactive ROS but may also be due to enzymatic processes as a result of increased activity of lipoxygenase (e.g., as a result of a pathogen invasion or wounding attack) [45,46]. Therefore, both enzymatic and non-enzymatic lipid peroxidation processes can lead to the formation of MDA. We found in our experiments that the NP1-Ag salt or NP2-Ag salt addition to the MS medium led to a decrease in the MDA content. Since the plants are grown in a sterile environment, it can be assumed that in the control plants grown on MS media free of NPs (C), the higher levels of MDA in stevia plantlets are a response only to the higher levels of non-enzymatic lipid peroxidation processes (oxidative stress). Besides being an indicator of damage to plant membranes, the MDA presence may be a sign of adaptation processes, as it serves as a signaling molecule to activate the regulatory genes involved in plant defense and reproduction [47,48]. Evidently, the low levels of MDA in the plantlets treated with NP1-Ag salt or NP2-Ag salt are due to the protective effect of MDA. Further biochemical, molecular, and genetic studies are needed to confirm these hypotheses. A decrease in the level of MDA in plant tissues under the influence of metal nanoparticles has been observed by several authors [49,50].

Proline is one of the osmoprotectants synthesized in plants in response to stress conditions. So far, it has been shown that under stress conditions, such as drought, salinization, high and low temperatures, heavy metals, mineral deficiency, UV, and biotic stress, proline concentration often increases to values several times higher than those observed under control conditions. Regardless of proline’s osmoprotective role, studies have shown that this molecule may also be involved in maintaining the redox balance in cells [51]. Whenever NF-1Ag salt and NF-2Ag salt were applied at the highest tested concentration (100 mg L^−1^), proline levels increased by two times compared to the control plantlets. In wheat plants, also at the higher AgNPs concentration tested, an almost threefold increase in this component has been reported [38,52].

One of the reaction products formed in the neutralization of the oxygen radical catalyzed by SOD is H_2_O_2_. Once formed, H_2_O_2_ is decomposed to O_2_ and H_2_O, products that the cell can easily eliminate. CAT is an antioxidant enzyme activated at high H_2_O_2_ levels, that can degrade H_2_O_2_. In *S. rebaudiana* plantlets, the increased values of SOD and CAT activities at concentrations of 1 mg L^−1^–50 mg L^−1^ NF-1Ag salt and NF-2Ag salt correlated with the low values of H_2_O_2_ at these same concentrations. The increased amount of H_2_O_2_ at 100 mg L^−1^ NF-1Ag salt and NF-2Ag salt indicated that the defense mechanisms were less activated at this concentration, and the decreased activity of SOD and CAT at 100 mg L^−1^ caused significant oxidative damage in the plant cells. When *Linum usitatissimum* L. has been treated with AgNPs, the increased values of antioxidant enzymes correspond with a decreased amount of H_2_O_2_ and malondialdehyde [53].

In the present study, the addition of the investigated organic nanofibers caused a reduction in the thiol group levels, which was more pronounced in the NF-1Ag salt treatment. With an increase in the applied concentrations of NF-Ag salts and a corresponding increase in Ag+ ions released by NF-Ag salts, which react with the thiol groups of the proteins, a decrease in the levels of thiol groups was recorded. According to Lok et al. [54], the amount of thiol groups in the cell can be reduced when silver ions are present in the medium. The authors have demonstrated the existence of sulfur in the cell, by energy-dispersive X-ray analysis, indicating that silver ions interact with thiol groups of proteins.

The elicitors are known to positively impact the production of biologically active secondary metabolites [55]. Nanoparticles were found to favor the biosynthesis of pharmacologically essential secondary metabolites in medicinal plants [56]. The experiment showed that the silver salt nanofibers (of monomer and dimer structures) negatively influenced the synthesis of stevioside and rebaudioside A in *S. rebaudiana* plantlets. In the plants treated with NF1-Ag salt, the decrease in the steviol glycoside content was more pronounced compared to NF2-Ag salt. This finding may be due to the silver ions’ higher content in the dimeric NF2-Ag salt compared to the monomeric NF1-Ag salt. Direct treatment with silver nanoparticles can act as a powerful amplifier of the transcriptional trigger of the genes of the steviol glycoside biosynthesis pathway that are able to positively control the production of steviol glycosides [57]. Various factors influence the activity of the elicitors, including their concentration, characteristics, size, stage of plant growth, physiochemical circumstances, and plant tissue cultures, such as callus culture, cell culture, and organ culture [58,59,60].

Despite the recorded decrease in the synthesis of stevioside and rebaudioside A, when NF1-Ag salt and NF2-Ag salt were added to the MS medium, an increase in the total soluble sugars was observed, being more pronounced in the treatment with nanofibers synthesized by the derivatives of the L-aspartic acid with a monomeric structure carrier of one silver ion, in the fourth studied concentration (NF1-Ag salt) in comparison to the treatment with the nanofibers synthesized by the derivatives of the L-aspartic acid with a dimeric molecular structure carrier of two silver ions. A decrease in the content of stevioside and rebaudioside A has been recorded in *S. rebaudiana* plantlets grown with nanofibers, formed from a derivative of the amino acid valine enriched with 1% and 2% colloidal silver (NF-1% Ag and NF-2% Ag), in comparison to control untreated plants, while the content of the total soluble sugar increased, particularly in the plants treated with NF-2% Ag [58]. The increased levels of soluble sugars are a consequence of the increased stress activity, caused by stress, of the enzymes involved in the induced hydrolysis of carbohydrates, leading to the conversion of these macromolecules into lower molecular weight compounds such as sugars and/or oligosaccharides, which can be used as a substrate in other metabolic pathways [61,62]. For example, glucose can be used as a substrate in the biosynthesis of secondary metabolites such as flavonoids in yellow lupine (*Lupinus luteus* L. cv. Polo) [63,64].

Mono- (CQA) and dicaffeoylquinic (DCQA) acids, as well as flavonoids, are valuable bioactive plant secondary metabolites with many health benefits (antiviral, hypoglycemic, hepatoprotective, and immunoprotective activities) due to their high antioxidant activity [65,66]. Chlorogenic (5-CQA) and 3,5-dicaffeoylquinic (3,5-DCQA) acids are principal components in *S. rebaudiana* leaves as well as their isomers 3-CQA, 4-CQA, 3,4-DCQA and 4,5-DCQA [65,66]. Quercetin 3-O-rhamnoside is one of the flavonoid compounds detected in stevia leaves, too [65,66]. The content of these secondary metabolites in *S. rebaudiana* is usually affected by the different ecological and climatic conditions [66]. Their in vitro production could be stimulated by adding various plant regulators, elicitors, etc. to the MS medium [67,68,69,70,71].

The present experiment showed that silver salt nanofibers with dimer structures positively (1 mg L^−1^) influenced the synthesis of CQA, DCQA, and quercetin 3-O-rhamnoside at *S. rebaudiana* plantlets and the silver salt nanofibers with monomer structures strongly reduced the production of phenolic compounds. This study further needs to investigate the effect of silver salt nanofibers on the metabolic pathways of secondary metabolites. In conclusion, the elicitors’ application seems to be a promising strategy to increase the metabolites of interest for human health.

## 4. Materials and Methods

### 4.1. Chemicals and Reagents

All solvents and other chemicals were of analytical grade. Ascorbate, guaiacol, hydrogen peroxide, DPPH, and ammonium molybdate were purchased from Merck (Darmstadt, Germany). Nitroblue tetrazolium, riboflavin, and methionine were purchased from Sigma (Jefferson, MO, USA). Stevioside, rebaudioside A, chlorogenic acid (5-CQA), 3,5-dicaffeoylquinic acid (3,5-DCQA), 4,5-dicaffeoylquinic acid (4,5-DCQA) and quercetin 3-O-rhamnoside (quercitrin, Qu 3-Rha) were purchased from Phytolab GmbH & Co. KG, Vestenbergsgreuth, Germany. Reagents and solvents for chemical synthesis were purchased from several companies—Alfa Aesar (Haverhill, MA, USA): Decanoic acid, DIEA [N,N-Diisopropylethylamine], Trifluoroacetic acid and silver nitrate (AgNO_3_); from Iris Biotech GmbH (Marktredwitz, Germany): Boc-L-Asp(OBzl)-OH, TBTU [O-(Benzotriazol-1-yl)-N,N,N′,N′-tetramethyluronium tetrafluoroborate]; from Merck (Darmstadt, Germany): DMF [Dimethylformamide], Ethylacetate, Hexane, NaHCO_3_, Citric acid, Chloroform, Methanol; Sigma-Aldrich (Jefferson, MO, USA): 5% Pd/C.

### 4.2. Chemical Nanofibers Synthesis

#### 4.2.1. Chemical Synthesis

**Figure 7:**
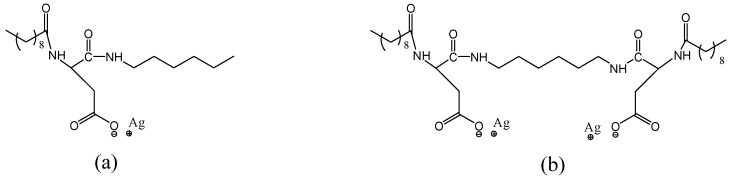
Molecular structures of the silver-organic salts used in our experiments: (**a**) NF1-Ag salt and (**b**) NF2-Ag salt.

Organic compounds used as carriers of silver ions have molecular structures, as presented in Figure 7.

#### 4.2.2. Chemical Synthesis of Both Compounds Has Been Accomplished in Similar Ways, Using the Corresponding Organic Compounds, Presented in Figure 8

**Figure 8:**
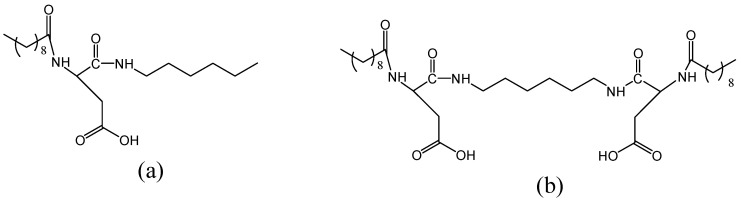
Organic compounds used for the preparation of silver salts: (**a**) dekanoyl-L-Asp-N-hexylamide and (**b**) N,N′-Bis(N-dekanoyl-L-Aspartyl)-diaminohexane.

Interactions of the organic compounds presented in Figure 8 with equivalent amounts of silver nitrate (AgNO_3_) in basic solutions gave their silver salt as presented in Figure 7. Organic compounds presented in Figure 7 have been obtained, according to procedures described previously starting from the amino acid protected form Boc-L-Asp(OBzl)-OH [72,73].

#### 4.2.3. Supramolecular Structures of Silver Salts Used

Silver salts were obtained for the first time in our work described here. It was found that they have very low solubility in almost all solvents. Only in acetic acid good solubility was estimated and such a solution was used for NMR analysis, while for structural determinations it was not applicable. Low solubility was found to occur in chloroform and DMSO. That is why to prepare samples for the AFM study, chloroform solutions were used. Chloroform is very volatile and evaporates quickly before it takes part in chemical reactions. Observations applying AFM on the nano and micrometer levels show that fibrils measuring several tenths of nanometers are formed by two salts as their inner structure.

### 4.3. Plant Material

*S. rebaudiana* Bert. seeds were purchased from the “Stevia-Paraguay” Company. Surface sterilization of the seeds was achieved by soaking them in 70% ethanol for 2 min, followed by 15% bleach solution (commercial bleach containing 4.85% sodium hypochlorite) for 15 min and triple rinsed in sterile distilled water each for 15 min. The seeds were germinated in vitro on MS media including vitamins supplemented with 3.0% sucrose, 7.0 g L^−1^ agar, 0.4 mg L^−1^ gibberellic acid, and 1.0 mg L^−1^ CaCl_2_ for three weeks. The in vitro culture conditions were maintained according to the protocol established by Zayova et al. [74]. Nodal explants were excised from the germinated seedlings and were cultured on MS without PGR for four weeks for shoot multiplication. For the experiments with nanofibers, shoot explants (1.5–2 cm) were cultured on MS media supplemented with various concentrations (1, 10, 50, 100 mg L^−1^) of two types of amino acid nanofibers enriched with Ag salt. The control plantlets were cultured on an MS nutrient medium without nanoparticles (C) [74]. Twenty shoot explants were placed on each medium variant, and each treatment was repeated twice. Cultures were incubated at 25 ± 2 °C under cool-white fluorescent light (Philips) with a 16 h photoperiod at an intensity of 40 mol m^−2^ s^−1^. After four weeks of cultivation, the plantlets were taken for analysis.

### 4.4. Biometrics

The average number of shoots per explant, shoot length, root length, and fresh weight of shoots and roots were determined. The potential micropropagation rate (MR) was calculated according to the following equation:

MR = SN × NN, (1)

where SN is the number of newly produced shoots and NN is the number of new internodes formed per shoot [75].

### 4.5. Antioxidants (Enzymatic and Non-Enzymatic)

The extraction of the enzymes with antioxidant activity (superoxide dismutase (SOD), catalase (CAT), ascorbate peroxidase (APX), and guaiacol peroxidase (GPO)) was performed according to Hristozkova et al. [76]. Total SOD (EC 1.15.1.1) activity [77], CAT (EC 1.11.1.6) activity [78], APX (EC 1.11.1.1) activity [79], and GPO (EC 1.11.1.7) activity [80] were determined spectrophotometrically on UV/VIS spectrophotometer (Shimadzu UV-1601, Tokyo, Japan). Soluble protein content was determined by using bovine serum albumin as a standard [81].

For the analysis of nonenzymatic antioxidant compounds, 0.3 g of dry material from each treatment was ground and suspended in 80% (*v*/*v*) methanol, and the extract was filtered. The filtrates were used for further investigations. Total phenolic content was measured spectrophotometrically using the Folin–Ciocalteau reagent and expressed as caffeic acid equivalents [82]. Total flavonoid content was measured spectrophotometrically and calculated using the standard curve of catechin [83]. The free radical scavenging activity was determined by an artificial color stable free radical DPPH (1,1-diphenyl-2-picrylhydrazyl) assay. The color changes (from deep violet to light yellow) were recorded at 517 nm by UV/VIS-spectrophotometer (Shimadzu, Tokyo, Japan) [84]. The ferric reducing antioxidant power assay (FRAP method) measures the reduction of the ferric tripyridyltriazine (Fe(III)-TPTZ) complex to the ferrous tripyridyltriazine (Fe(II)-TPTZ) by antioxidants at low pH [85]. The content of water-soluble (WS-AOM) and lipid-soluble (LS-AOM) metabolites with antioxidant potential was measured spectrophotometrically and expressed as equivalents of ascorbate and α-tocopherol, respectively [86]. This assay was based on the formation of a green-colored phosphomolybdenum complex due to the reduction of Mo (VI) to Mo (V) by the samples.

### 4.6. Stress Markers Estimation

For the determination of the content of hydrogen peroxide (H_2_O_2_), malondialdehyde (MDA), proline, and free thiol group-containing compounds (SH-), 300 mg fresh leaf material was homogenized with 0.1% (*w*/*v*) trichloroacetic acid. Hydrogen peroxide content was measured spectrophotometrically according to a protocol described by Alexieva et al. [87]. Malondialdehyde content was determined according to Kramer et al. [88], as a thiobarbituric acid-reagent product, using the extinction coefficient of 155 mM^−1^ cm^−1^. The quantitative analysis of free proline was performed according to Bates et al. [89], after a reaction with acidic ninhydrin with the formation of a red-colored compound, and absorbance was read at 520 nm. For the determination of free thiol group-containing compound content, 40 µL supernatant was incubated with 150 µL Ellman’s reagent for 10 min at room temperature and the absorbance was read at 412 nm [90].

### 4.7. Soluble Sugar Analyses

Total soluble sugars were determined by the phenol–sulphuric acid method, according to the procedure described by Ashwell [91].

### 4.8. Stevioside, Rebaudioside A, and Soluble Sugar Analyses

For HPLC analysis, each sample was prepared as follows: 50 mg of dried and powdered leaves were extracted with 5 mL of distilled water at 40 °C in an ultrasonic bath for 30 min. The extracts were centrifuged and filtered. The filtrates were transferred to a volumetric flask and made up to 5 mL with distilled water. The extracts were then processed with solid phase extraction (SPE) cartridges filled with C_18_ sorbents (Superclean^TM^ LC-18, 500 mg, 3 mL, Supelco, Bellefonte, PA, USA) according to the protocol described by Bergs et al. [92]. Before injection, samples were filtered through a 0.22 mm membrane filter.

The HPLC analysis was peIformed on Shimadzu Nexera-i LC-2040C 3D Plus liquid chromatograph equipped with a photodiode array detector (Shimadzu, Tokyo, Japan), analytical column Inertsil NH_2_ (4.0 × 150 mm, 3 µm) (GL Sciences, Tokyo, Japan), wavelength 210 nm, mobile phase CH_3_CN:H_2_O (80:20, *v*/*v*) in an isocratic mode for 30 min, at a temperature of 40 °C, a flow rate of 0.8 mL min^−1^ and injection volume of 4 µL. Retention times (Rt) and UV spectra were compared with those of pure standards. Calibration curves at different concentrations were made from stevioside (0.0625–1.00 mg mL^−1^, R^2^—0.9998) and rebaudioside A (0.0625–1.00 mg mL^−1^, R^2^—0.9998) as standards. All determinations were done in triplicate. The contents were expressed in mg/g DM.

### 4.9. Determination of Mono- and Dicaffeoylquinic Acids and Quercetin-3-O-rhamnoside

For HPLC analysis, each sample was prepared as follows: 150 mg of dried and powdered leaves were extracted twice with 5 mL of methanol at 25 °C in an ultrasonic bath for 30 min. The extracts were centrifuged and filtered. The filtrates were transferred to a volumetric flask and made up to 10 mL with methanol. One milliliter of the extracts was passed through solid phase extraction (SPE) cartridges filled with C_18_ sorbents (Chromabond^®^, 200 mg, 3 mL, Machery-Nagel, GMBH&Co., KG, Duren, Germany) to remove chlorophylls.

The HPLC analysis was performed on Shimadzu Nexera-i LC-2040C 3D Plus liquid chromatograph equipped with a photodiode array detector (Shimadzu, Tokyo, Japan), analytical column Force C18 (150 × 4.6 mm, 3 µm) (Restek, Bellefonte, PA, USA) and its temperature was maintained at 40 °C. Gradient elution was carried out with a mixture of two solvents: (A) 0.1% (*v*/*v*) of formic acid in water and (B) 0.1% (*v*/*v*) of formic acid in acetonitrile. The following gradient program was performed: 0 min, 10% B; 5 min, 15% B; 10–15 min, 18% B; 18–21 min, 25% B; 25–27 min, 60% B; 27.01–29 min, 80% B, 30–35 min, 10% B. The flow rate was 0.6 mL/min and the injected volume was equal to 2 μL. The extracts and mobile phases were filtered through a 0.22 mm membrane filter and then degassed by an ultrasonic bath prior to use. The runs were monitored at the following wavelengths: phenolic acids at 325 nm and quercetin 3-O-rhamnoside at 350 nm. Retention times (Rt) and UV spectra were compared with those of pure standards. Calibration curves at different concentrations were made from chlorogenic acid (0.027–0.423 mg mL^−1^, R^2^—0.9996), 3,5-dicaffeoylquinic acid (0.026–0.410 mg mL^−1^, R^2^—0.9995), 4,5-dicaffeoylquinic acid (0.022–0.363 mg mL^−1^, R^2^—0.9997), and quercetin 3-O-rhamnoside (0.007–0.057 mg mL^−1^, R^2^—0.9994) as standards. The quantities of neochlorogenic acid (3-CQA) and cryptochlorogenic acid (4-CQA) were assessed from peak areas and calculated as equivalents of chlorogenic acid (5-CQA), and 3,4-dicaffeoylquinic acid (3,4-DCQA) as equivalents of 3,5-DCQA. All determinations were done in triplicate. The contents were expressed in mg/g DM.

### 4.10. Statistical Analysis

The data were statistically processed by one-way ANOVA analyses of variance for comparison of means. Significant differences were calculated according to Fisher’s least significance difference (LSD) test at the 5% significance level using a statistical software package (Statgraphics Plus, version 5.1 for Windows, (1994) Statistical Graphics Corporation, Warrenton, VA, USA). Principal component analysis (PCA) was performed using SIMCA 17 (USA).

## 5. Conclusions

In conclusion, our research discovered the optimal concentration of both investigated nanofibers for the in vitro propagation of *S. rebaudiana*. With an increase in the concentration of nanofibers' NF1-Ag salt from 1 to 50 mg L^−1^ added into the MS medium, a significantly raised shoot height, number, and biomass accumulation of *S. rebaudiana* Bert. plantlets were observed. The monitored magnification in the level of the micropropagation rate was mainly due to the increased internode number. With a further increase in the concentration (100 mg L^−1^) of the studied nanofibers, a decrease in the growth parameters was observed. The treatment with the NF2-Ag salt did not affect significantly the height and biomass accumulation of the shoots; only the number of shoots increased. In addition, an increase in the number of internodes and a decrease in the internodes’ length was recorded. This, in turn, led to an increase in the potential micropropagation rate, which is determined by the number of internodes and the internodes’ length.

Despite the higher levels of the parameters characterizing the growth of the *S. rebaudiana* plantlets when NF1-Ag salt was added to the MS medium, the content of stevioside, rebaudioside A, mono- (CQA), and dicaffeoylquinic (DCQA) acids, as well as flavonoid quercetin 3-rhamnoside, was significantly reduced compared to the control plants. As opposed to NF1-Ag salt, the treatment with NF2-Ag salt has a positive effect on the content of stevioside, rebaudioside A, mono- (CQA), and dicaffeoylquinic (DCQA) acids, and Quercetin 3-O-Rhamnoside.

Further studies are needed to evaluate the influence of the studied nanofibers NF1-Ag salt and NF2-Ag salt on plantlets’ soil adaptation. It is important to know what the reaction of the obtained in vitro morphotypes at different abiotic and biotic stress conditions will be. Our findings may open a new window for using the studied nanofibers to obtain *S. rebaudiana* plants rich in steviol glycosides.

## Figures and Tables

**Table 1:** Morphological parameters of *S. rebaudiana* plantlets grown on NF-free MS media, on MS media supplemented with nanofibers synthesized by the derivatives of the L-aspartic acid with a monomeric molecular structure carrier of one silver ion (NF1-Ag salt), in concentrations 1, 10, 50 and 100 mg L^−1^.

	MS	MS + NF1-Ag-Salt				
Treatments		1 mg L^−1^	10 mg L^−1^	50 mg L^−1^	100 mg L^−1^	LSD
FW shoot (g plant^−1^)	0.115 ± 0.001 ^a^	0.311 ± 0.032 ^c^	0.374 ± 0.019 ^d^	0.389 ± 0.020 ^d^	0.237 ± 0.012 ^b^	0.026
DW shoot (g plant^−1^)	0.018 ± 0.001 ^a^	0.043 ± 0.002 ^b^	0.076 ± 0.004 ^d^	0.066 ± 0.003 ^c^	0.020 ± 0.001 ^a^	0.005
Shoot height (cm explant^−1^)	5.97 ± 0.30 ^bc^	10.14 ± 0.51 ^d^	6.39 ± 0.32 ^c^	5.48 ± 0.28 ^b^	2.01 ± 0.10 ^a^	0.595
Shoot number (explant^−1^)	1.00 ± 0.05 ^a^	1.45 ± 0.07 ^b^	1.99 ± 1.48 ^c^	3.07 ± 0.154 ^b^	1.44 ± 0.07 ^b^	0.17
Root initiation (%)	0.00 ± 0.00 ^a^	8.00 ± 0.40 ^c^	5.13 ± 0.26 ^b^	5.17 ± 0.26 ^b^	5.26 ± 0.26 ^b^	0.49
No internodes (plant^−1^)	2.50 ± 0.13 ^b^	3.90 ± 0.20 ^c^	5.10 ± 0.26 ^de^	4.90 ± 0.25 ^d^	2.03 ± 0.10 ^a^	0.35
Internode length (plant^−1^)	1.50 ± 0.08 ^c^	2.60 ± 0.13 ^d^	1.25 ± 0.06 ^b^	1.08 ± 0.05 ^a^	1.01 ± 0.05 ^a^	0.14
Micropropagation rate MR	2.50 ± 0.13 ^a^	5.66 ± 0.28 ^b^	10.15 ± 0.51 ^c^	15.04 ± 0.75 ^d^	2.92 ± 0.15 ^a^	0.78

Values are means ± SE, *n* = 20; different letters indicate significant differences assessed by the Fisher LSD test (*p* ≤ 0.05) after performing ANOVA one-way analysis. We use the letter “a” for the lowest data value and ascend to the next letters for higher data value.

**Table 2:** Morphological parameters of *S. rebaudiana* plantlets grown on NF-free MS media and on MS media supplemented with nanofibers synthesized by the derivatives of the L-aspartic acid with a dimeric molecular structure carrier of two silver ions (NF2-Ag-salt), in concentrations 1, 10, 50, and 100 mg L^−1^.

	MS	NF2-Ag Salt				
		1 mg L^−1^	10 mg L^−1^	50 mg L^−1^	100 mg L^−1^	LSD
FW shoot (g plant^−1^)	0.115 ± 0.006 ^bc^	0.121 ± 0.006 ^c^	0.105 ± 0.005 ^b^	0.139 ± 0.007 ^d^	0.061 ± 0.003 ^a^	0.013
DW shoot (g plant^−1^)	0.018 ± 0.001 ^b^	0.013 ± 0.001 ^a^	0.015 ± 0.001 ^b^	0.014 ± 0.001 ^a^	0.009 ± 0.001 ^a^	0.005
Shoot height (cm explant^−1^)	5.97 ± 0.30 ^c^	6.46 ± 0.32 ^d^	5.10 ± 0.26 ^b^	4.80 ± 0.24 ^b^	2.16 ± 0.12 ^a^	0.47
Shoot number (explant^−1^)	1.00 ± 0.05 ^a^	1.40 ± 0.07 ^bc^	1.39 ± 0.07 ^bc^	1.49 ± 0.08 ^c^	1.30 ± 0.07 ^b^	0.12
Root initiation (%)	0.00 ± 0.00 ^a^	37.50 ± 1.88 ^bc^	62.50 ± 3.13 ^d^	34.10 ± 1.71 ^b^	40.00 ± 2.00 ^c^	3.66
No internodes (plant^−1^)	2.50 ± 0.13 ^b^	4.25 ± 0.21 ^e^	3.85 ± 0.19 ^d^	3.45 ± 0.17 ^c^	1.59 ± 0.08 ^a^	0.30
Internode length (plant^−1^)	1.50 ± 0.08 ^b^	1.52 ± 0.08 ^b^	1.32 ± 0.09 ^a^	1.38 ± 0.07 ^ab^	1.36 ± 0.07 ^a^	0.14
Micropropagation rate MR	2.50 ± 0.13 ^b^	5.95 ± 0.30 ^d^	5.35 ± 0.27 ^c^	5.14 ± 0.26 ^c^	2.07 ± 0.10 ^a^	0.41

Values are means ± SE, *n* = 20; different letters indicate significant differences assessed by the Fisher LSD test (*p* ≤ 0.05) after performing ANOVA one-way analysis. We use the letter “a” for the lowest data value and ascend to the next letters for higher data value.

**Table 3:** Content of mono- (CQA) and dicaffeoylquinic (DCQA) acids and quercetin-3-O-rhamnoside (Qu-3-Rha) (mg g^−1^ DW) in the *Stevia rebaudiana* plantlets in vitro propagated on MS media free of NP, and on MS media supplemented with various concentrations (1, 10, 50, 100 mg L^−1^) of nanofibers synthesized by the derivatives of the L-aspartic acid with a monomeric (NF1-Ag salt) or dimeric molecular structure (NF2-Ag salt) carrier of silver ions.

	3-CQA	5-CQA	4-CQA	3,5-DCQA	3,4-DCQA	4,5-DCQA	Qu-3-Rha	Total QA
C	0.35 ± 0.01 ^a^	4.27 ± 0.04 ^a^	0.51 ± 0.01 ^a^	4.03 ± 0.07 ^a^	0.28 ± 0.02 ^a^	1.97 ± 0.03 ^a^	0.31 ± 0.02 ^a^	11.41 ± 0.13 ^a^
NF1-Ag salt								
1 mg L^−1^	0.06 ± 0.00 ^b^	0.40 ± 0.01 ^b^	0.10 ± 0.01 ^b^	0.34 ± 0.01 ^b^	0.07 ± 0.01 ^b^	0.28 ± 0.02 ^b^	0.10 ± 0.00 ^b^	1.25 ± 0.04 ^b^
10 mg L^−1^	0.04 ± 0.00 ^c^	0.26 ± 0.01 ^c^	0.06 ± 0.015 ^c^	0.32 ± 0.01 ^b^	0.11 ± 0.01 ^c^	0.21 ± 0.01 ^c^	0.08 ± 0.01 ^c^	0.99 ± 0.02 ^c^
50 mg L^−1^	0.06 ± 0.00 ^b^	0.50 ± 0.01 ^d^	0.08 ± 0.01 ^b^	0.48 ± 0.02 ^c^	0.26 ± 0.03 ^a^	0.39 ± 0.01 ^b^	0.11 ± 0.01 ^b^	1.67 ± 0.03 ^d^
100 mg L^−1^	0.04 ± 0.00 ^c^	0.46 ± 0.03 ^d^	0.03 ± 0.01 ^d^	0.50 ± 0.02 ^c^	0.19 ± 0.01 ^d^	0.171 ± 0.004 ^d^	0.06 ± 0.00 ^d^	1.40 ± 0.03 ^e^
NF2-Ag salt								
1 mg L^−1^	0.27 ± 0.01 ^d^	4.80 ± 0.081 ^e^	0.58 ± 0.02 ^e^	6.84 ± 0.05 ^d^	0.57 ± 0.01 ^e^	2.76 ± 0.07 ^e^	0.61 ± 0.01 ^e^	15.81 ± 0.01 ^f^
10 mg L^−1^	0.15 ± 0.01 ^e^	2.23 ± 0.027 ^f^	0.39 ± 0.01 ^f^	4.13 ± 0.04 ^a,e^	0.43 ± 0.01 ^f^	1.81 ± 0.01 ^f^	0.43 ± 0.01 ^f^	9.15 ± 0.106 ^g^
50 mg L^−1^	0.27 ± 0.01 ^d^	3.26 ± 0.04 ^g^	0.53 ± 0.02 ^a^	4.80 ± 0.02 ^f^	0.44 ± 0.02 ^f^	2.17 ± 0.06 ^g^	0.50 ± 0.01 ^g^	11.47 ± 0.18 ^a^
100 mg L^−1^	0.17 ± 0.02 ^e^	2.33 ± 0.05 ^h^	0.26 ± 0.02 ^g^	4.17 ± 0.05 ^a,e^	0.40 ± 0.01 ^g^	1.77 ± 0.08 ^f^	0.32 ± 0.01 ^a^	9.10 ± 0.17 ^g^

Data from all the measurements are the mean of three replicates ± standard deviation. Values with different letters in the columns are significantly different (*p* < 0.05, *t*-test); Total QA—sum of all identified CQA and DCQA.

**Table 4:** Pearson’s correlation coefficients (r) between phenolic content (TPC, TFC, and individual compounds) and antioxidant activities (DPPH, and FRAP).

	TPC	3-CQA	5-CQA	4-CQA	3,5-DCQA	3,4-DCQA	4,5-DCQA	Qu-3-Rha
DPPH	0.971 **	0.507	0.606	0.712 *	0.827 **	0.847 **	0.808 **	0.885 **
FRAP	0.629	0.721 *	0.768 *	0.723 *	0.823 **	0.791 *	0.828 **	0.743 *

Significant correlation with *p* < 0.05 (*) and *p* < 0.001 (**).

## Data Availability

Data available in a publicly accessible repository.

## Supplementary Materials

The following supporting information can be downloaded at: https://www.mdpi.com/article/10.3390/plants12203574/s1, Figure S1. *Stevia rebaudiana* plantlets grown on NF-free MS medium (A); on MS medium supplemented with nanofibers synthesized by the derivatives of the L-aspartic acid with a monomeric structure, carrier of one silver ion (NF1-Ag-salt), in concentrations 1, 10, 50 and 100 mg L^−1^ (B, C, D, E). Figure S2. *Stevia rebaudiana* plantlets grown on NF-free MS medium (A) and on MS medium supplemented nanofibers synthesized by the derivatives of the L-aspartic acid with a dimeric molecular structure, carrier of two silver ions (NF2-Ag salt), in concentrations 1, 10, 50 and 100 mg L^−1^ (B, C, D, E). Figure S3. AFM - pictures of NF1-Ag salt. Fibrils arranged in parallel are observed, with a diameter of a few tens of nanometers. Figure S4. AFM - pictures of NF2-Ag salt. Disordered filaments are observed, with a thickness of about 20–30 nanometers. Figure S5. HPLC chromatograms (210 nm) of standard mixture of stevioside and rebaudioside A and different extracts of *Stevia rebaudiana* plantlets in vitro propagated on MS medium free of NP, and on MS medium supplemented with 1 mg L^−1^ of nanofibers synthesised by the derivatives of the L-aspartic acid with a monomeric (NF1-Ag salt) or dimeric molecular structure (NF2-Ag salt), carrier of silver ions. Figure S6. HPLC chromatograms (325 nm) of standard mixture of 5-CQA, 3,5-DCQA and 4,5-DCQA and different extracts of *Stevia rebaudiana* plantlets in vitro propagated on MS medium free of NP, and on MS medium supplemented with 1 mg L^−1^ of nanofibers synthesised by the derivatives of the L-aspartic acid with a monomeric (NF1-Ag salt) or dimeric molecular structure (NF2-Ag salt), carrier of silver ions. Figure S7. HPLC chromatograms (350 nm) of standard quercetin-3-O-rhamnoside (Qu-3-Rha) and different extracts of *Stevia rebaudiana* plantlets in vitro propagated on MS medium free of NP, and on MS medium supplemented with 1 mg L^−1^ of nanofibers synthesised by the derivatives of the L-aspartic acid with a monomeric (NF1-Ag salt) or dimeric molecular structure (NF2-Ag salt), carrier of silver ions. Table S1. MDA, -SH, H_2_O_2_, Proline. Table S2. SOD, CAT, APX, GPO. Table S3. Morphol. parameters NF2. Table S4. WS-,LS-, Flav., Phen., DPPH, FRAP. Table S5. Steviosides. Table S6. Morphol. parameters.
