# Supplementary material for: Influence of the Abiotic Elicitors Ag Salts of Aspartic Acid Derivatives, Self-Organized in Nanofibers with Monomeric and Dimeric Molecular Structures, on the Antioxidant Activity and Stevioside Content in Micropropagated Stevia rebaudiana Bert
Source: Plants (Basel). 2023 Oct 14;12(20):3574. doi: 10.3390/plants12203574 (PMC10610515; doi:10.3390/plants12203574)
Supplement: Supplementary file 1 [file plants-12-03574-s001.zip › Figures S1-S7.pdf]

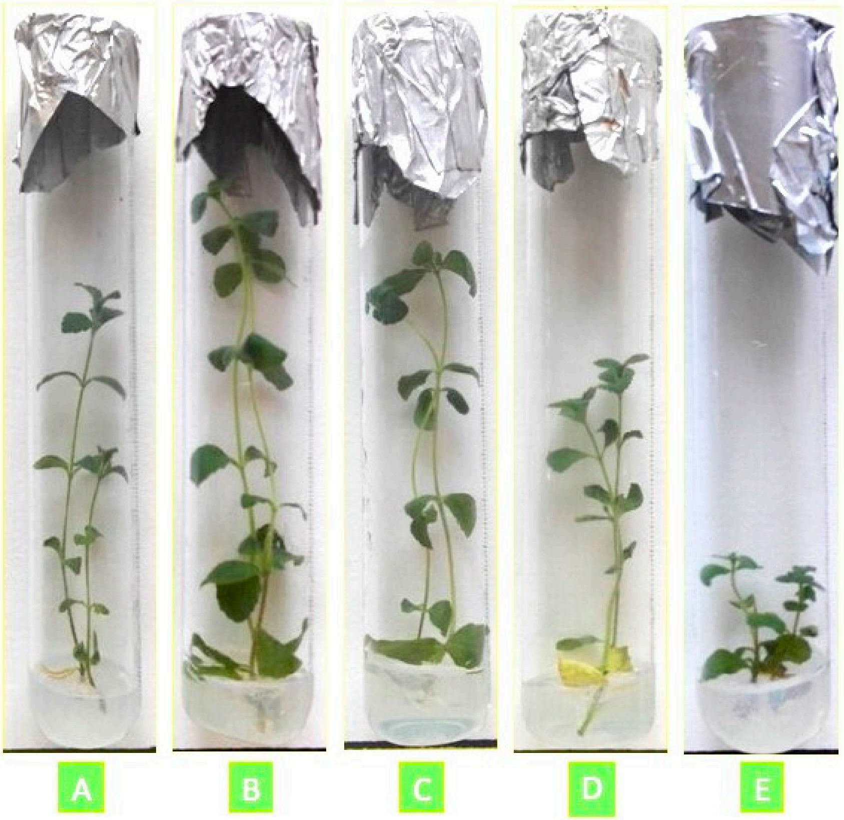

Figure S1. *Stevia rebaudiana* plantlets grown on NF-free MS medium (**A**); on MS medium supplemented with nanofibers synthesized by the derivatives of the L-aspartic acid with a monomeric structure, carrier of one silver ion (NF1-Ag-salt), in concentrations 1, 10, 50 and 100 mg L^{-1} (**B, C, D, E**)

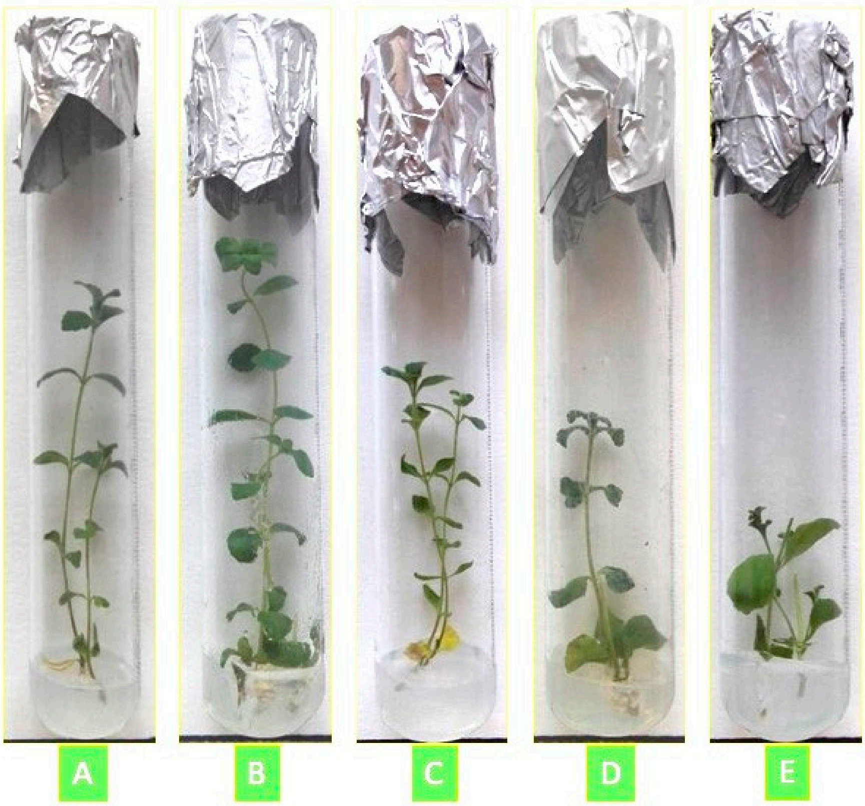

Figure S2. *Stevia rebaudiana* plantlets grown on NF-free MS medium (**A**) and on MS medium supplemented nanofibers synthesized by the derivatives of the L-aspartic acid with a dimeric molecular structure, carrier of two silver ions (NF2-Ag salt), in concentrations 1, 10, 50 and 100 mg L^{-1} (**B, C, D, E**)

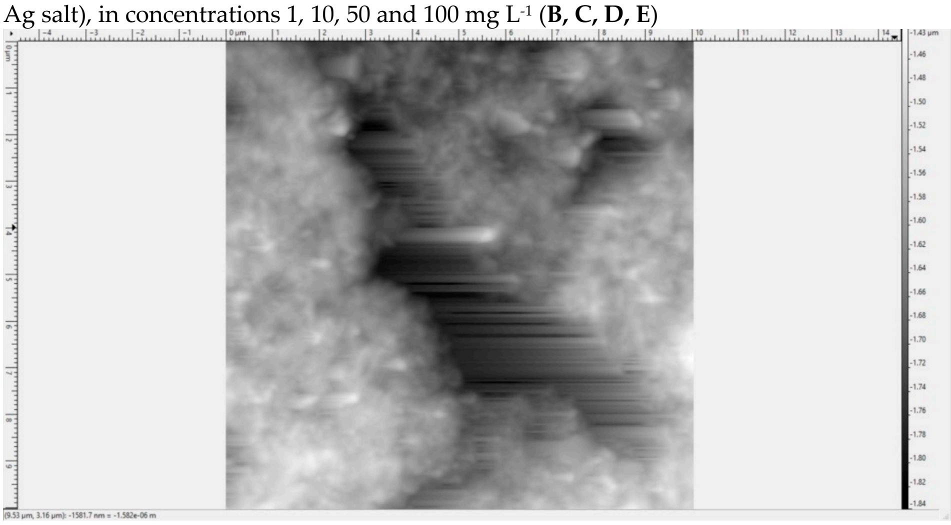

Figure S3. AFM - pictures of NF1-Ag salt. Fibrils arranged in parallel are observed, with a diameter of a few tens of nanometers.

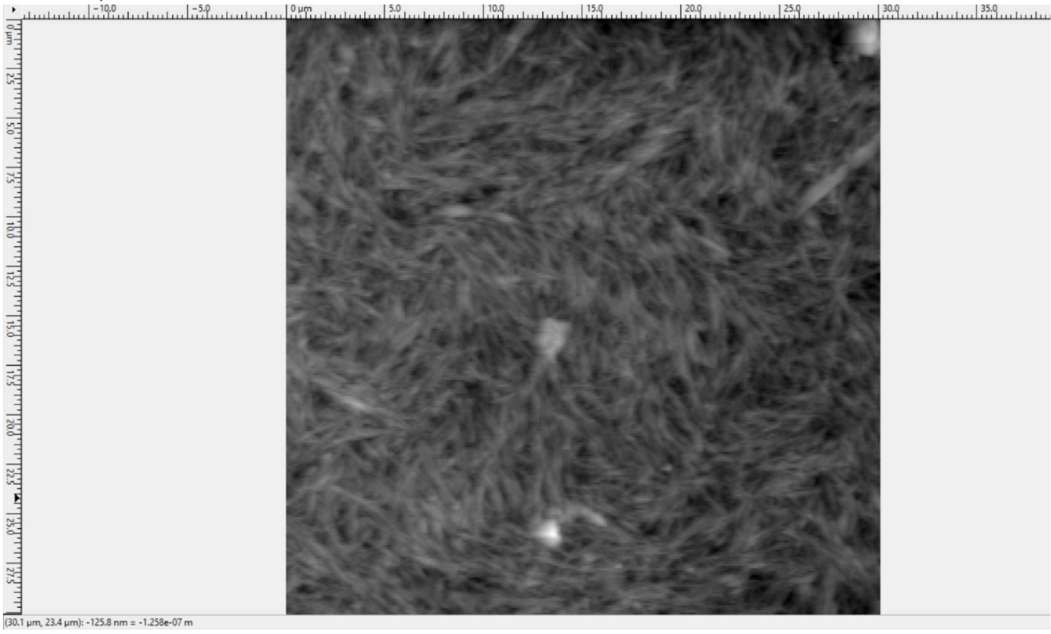

Figure S4. AFM - pictures of NF2-Ag salt. Disordered filaments are observed, with a thickness of about 20-30 nanometers.

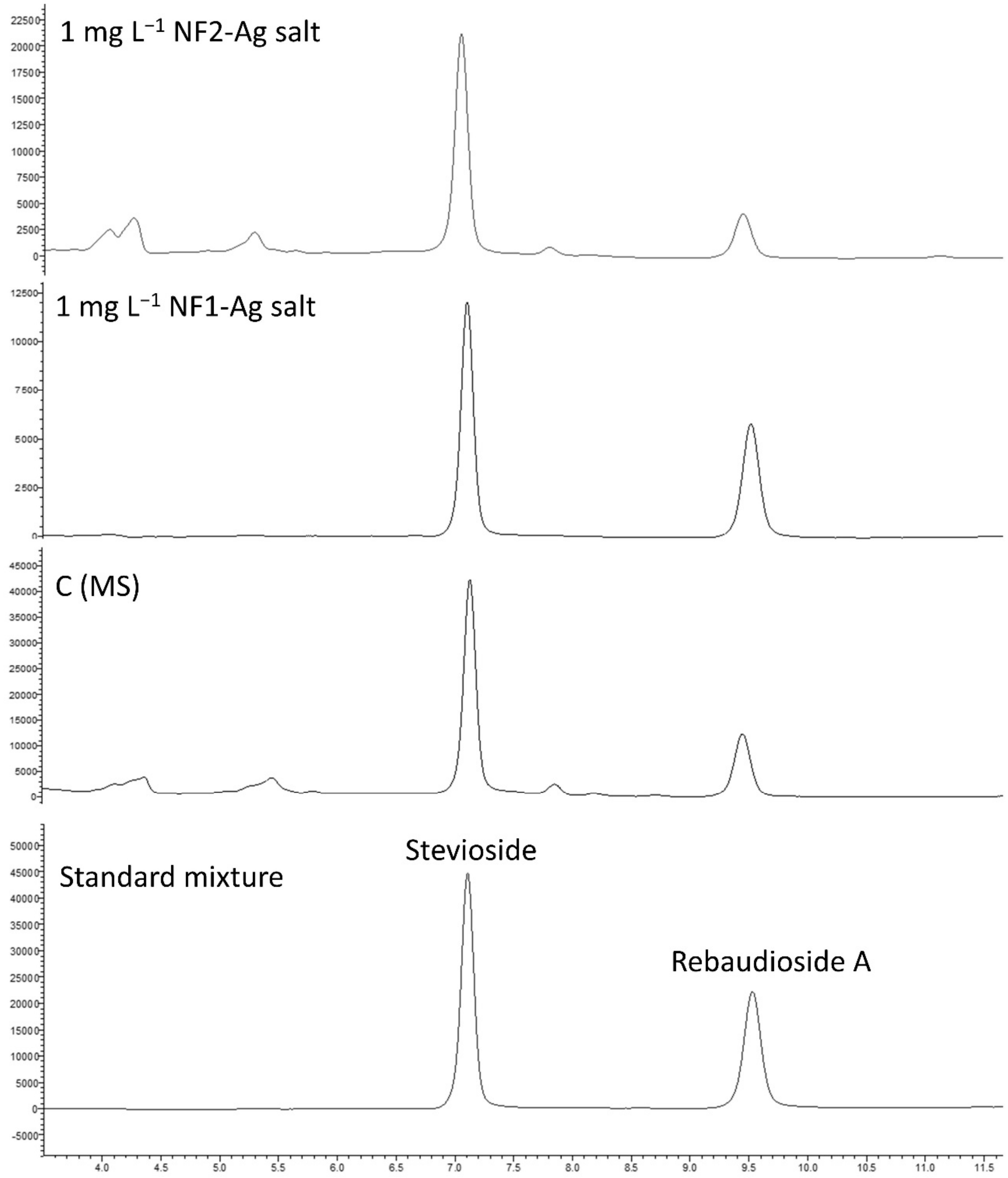

Figure S5. HPLC chromatograms (210 nm) of standard mixture of stevioside and rebaudioside A and different extracts of *Stevia rebaudiana* plantlets *in vitro* propagated on MS medium free of NP, and on MS medium supplemented with 1 mg L^{-1} of nanofibers synthesised by the derivatives of the L-aspartic acid with a monomeric (NF1-Ag salt) or dimeric molecular structure (NF2-Ag salt), carrier of silver ions

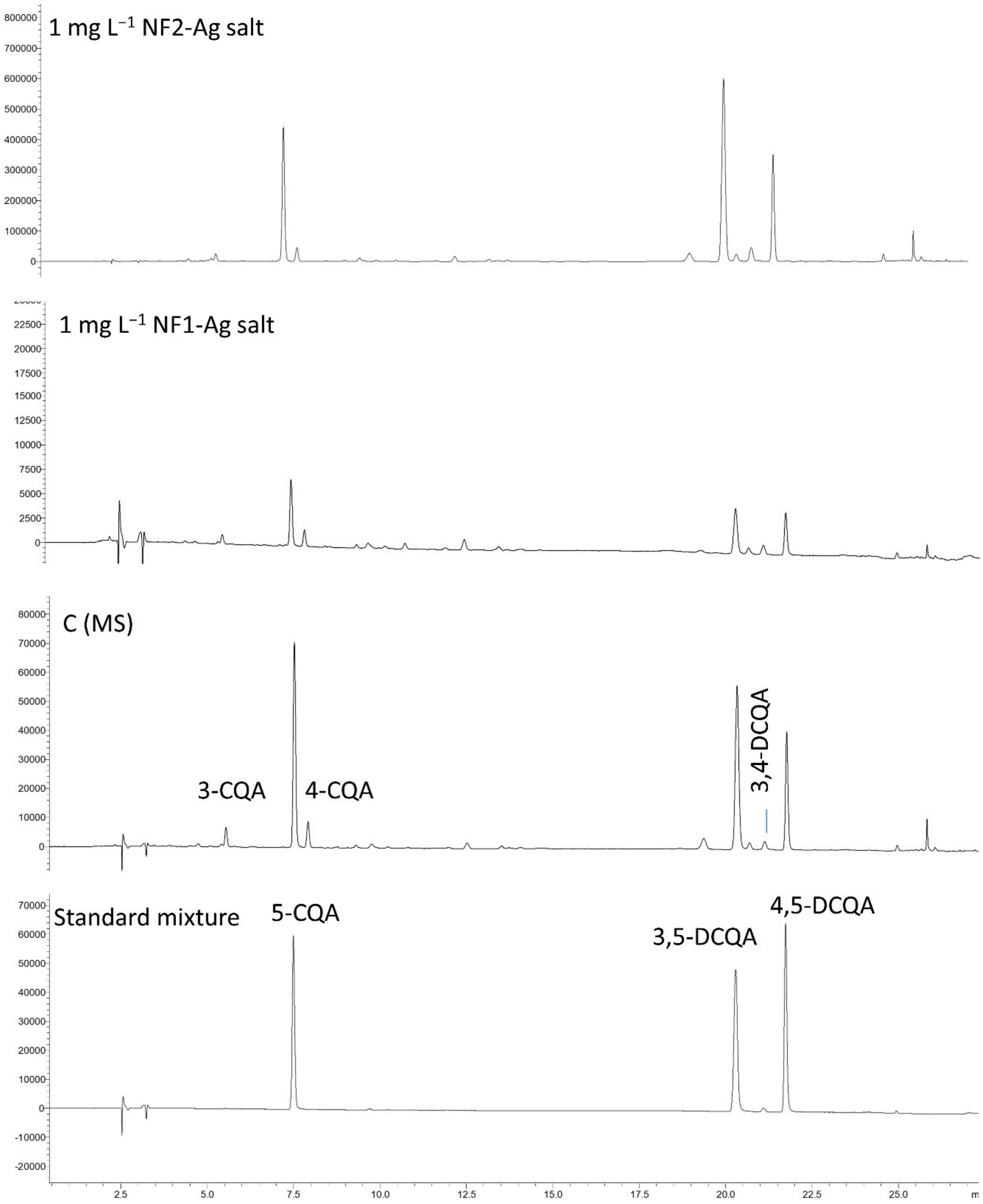

Figure S6. HPLC chromatograms (325 nm) of standard mixture of 5-CQA, 3,5-DCQA and 4,5-DCQA and different extracts of *Stevia rebaudiana* plantlets *in vitro* propagated on MS medium free of NP, and on MS medium supplemented with 1 mg L^{-1} of nanofibers synthesised by the derivatives of the L-aspartic acid with a monomeric (NF1-Ag salt) or dimeric molecular structure (NF2-Ag salt), carrier of silver ions

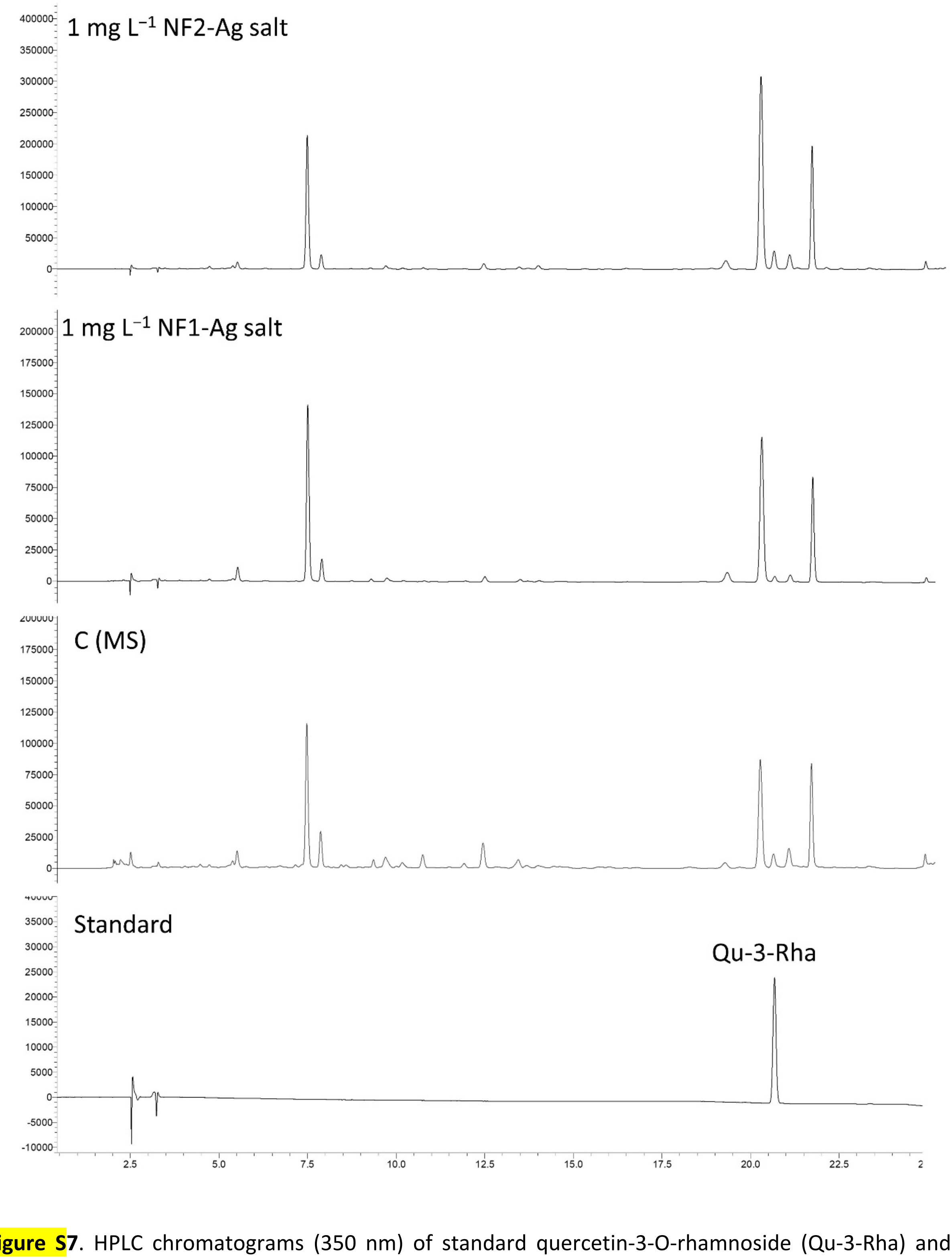

Figure S7. HPLC chromatograms (350 nm) of standard quercetin-3-O-rhamnoside (Qu-3-Rha) and different extracts of *Stevia rebaudiana* plantlets *in vitro* propagated on MS medium free of NP, and on MS medium supplemented with 1 mg L^{-1} of nanofibers synthesised by the derivatives of the L-aspartic acid with a monomeric (NF1-Ag salt) or dimeric molecular structure (NF2-Ag salt), carrier of silver ions
