# Supplementary material for: Influence of the Abiotic Elicitors Ag Salts of Aspartic Acid Derivatives, Self-Organized in Nanofibers with Monomeric and Dimeric Molecular Structures, on the Antioxidant Activity and Stevioside Content in Micropropagated Stevia rebaudiana Bert
Source: Plants (Basel). 2023 Oct 14;12(20):3574. doi: 10.3390/plants12203574 (PMC10610515; doi:10.3390/plants12203574)
Supplement: Supplementary file 1 [file plants-12-03574-s001.zip › Table S1. MDA, -SH, H2O2, Proline.pdf]

MDA-NF1 Ag salt					
5	14.08	x			
4	22.45		x		
3	23.11		x		
2	24.53		x		
1	37.91			x	
LSD	2.332				
MDA-NF2 Ag salt					
3	33.54	x			
2	34.25	x			
4	35.19	x	x		
5	37.84		x		
1	37.91		x		
LSD	3.257				

-SH NF1 Ag salt					
5	0.15	x			
4	0.11	x			
3	0.34		x		
2	0.46			x	
1	1.39				x
LSD	0.062				
-SH NF2 Ag salt					
5	0.79	x			
2	0.96		x		
4	0.99		x	x	
3	1.08			x	
1	1.39				x
LSD	0.096				

H_2O_2-NF1 Ag salt					
3	6.33	x			
2	10.15		x		
4	15.91			x	
5	32.91				x
1	33.37				x
LSD	2.077				
H_2O_2-NF2 Ag salt					
2	4.78	x			
3	5.06	x			
4	9.98		x		
1	33.37			x	
5	33.71			x	
LSD	1.995				

Proline-NF1 Ag salt					
4	2.06	x			
3	2.82		x		
2	3.6			x	
1	3.65			x	
5	7.11				x
LSD	0.385				
Proline-NF2 Ag salt					
4	2.903	x			
3	3.503		x		
1	3.65		x		
2	3.91		x		
5	8.2			x	
LSD	0.44				
