# Supplementary material for: Influence of the Abiotic Elicitors Ag Salts of Aspartic Acid Derivatives, Self-Organized in Nanofibers with Monomeric and Dimeric Molecular Structures, on the Antioxidant Activity and Stevioside Content in Micropropagated Stevia rebaudiana Bert
Source: Plants (Basel). 2023 Oct 14;12(20):3574. doi: 10.3390/plants12203574 (PMC10610515; doi:10.3390/plants12203574)
Supplement: Supplementary file 1 [file plants-12-03574-s001.zip › Table S2. SOD, CAT, APX, GPO.pdf]

SOD-NF1 Ag salt						
1	60.41	x				
4	173.93		x			
5	192.82			x		
2	224.0				x	
3	286.89					x
LSD	18.352					
SOD-NF2 Ag salt						
1	60.41	x				
5	238.21		x			
2	239.2		x			
3	302.72			x		
4	380.27				x	
LSD	24.199					

CAT-NF1 Ag salt					
5	9.07	x			
1	9.8	x	x		
2	10.76		x		
4	12.67			x	
3	13.34			x	
LSD	1.023				
CAT-NF2 Ag salt					
1	9.8	x			
5	10.68	x			
4	12.87		x		
3	13.53		x		
2	15.19			x	
LSD	1.142				

APX-NF1 Ag salt						
4	0.103	x				
5	0.19		x			
2	0.23			x		
3	0.27				x	
1	0.39					x
LSD	0.022					
APX-NF2 Ag salt						
1	0.39	x				
4	0.503		x			
5	0.51		x			
3	0.52		x			
2	0.57			x		
LSD	0.05					

GPX-NF1 Ag salt						
5	0.047	x				
4	0.51		x			
2	1.03			x		
1	1.37				x	
3	1.62					x
LSD	0.099					
GPX-NF2 Ag salt						
5	0.05	x				
4	0.903		x			
1	1.37			x		
3	1.98				x	
2	2.8					x
LSD	0.156					
