# Supplementary material for: Influence of the Abiotic Elicitors Ag Salts of Aspartic Acid Derivatives, Self-Organized in Nanofibers with Monomeric and Dimeric Molecular Structures, on the Antioxidant Activity and Stevioside Content in Micropropagated Stevia rebaudiana Bert
Source: Plants (Basel). 2023 Oct 14;12(20):3574. doi: 10.3390/plants12203574 (PMC10610515; doi:10.3390/plants12203574)
Supplement: Supplementary file 1 [file plants-12-03574-s001.zip › Table S3. Morphol. parameters NF2.pdf]

FWshoot NF2 Ag salt							
5	0.061	x					
3	0.105		x				
1	0.115		x	x			
2	0.121			x			
4	0.139				x		
LSD	0.01						
DWshoot NF2 Ag salt							
5	0.009	x					
2	0.013		x				
4	0.014		x	x			
3	0.015			x			
1	0.018				x		
LSD	0.002						
Shoot height NF2 Ag salt							
5	2.16	x					
4	4.8		x				
3	5.1		x				
1	5.97			x			
2	6.46				x		
LSD	0.466						
Shoot number NF2 Ag salt							
1	1.0	x					
5	1.3		x				
3	1.39		x	x			
2	1.4		x	x			
4	1.49			x			
LSD	0.121						
Root init. NF2 Ag salt							
1	0.0	x					
4	34.1		x				
2	37.5		x	x			
5	40.0			x			
3	62.5				x		
LSD	3.656						
No internodes NF2 Ag salt							
5	1.59	x					

1	2.5		x				
4	3.45			x			
3	3.85				x		
2	4.25					x	
LSD	0.298						
Internodes length NF2 Ag salt							
3	1.32	x					
5	1.36	x					
4	1.38	x	x				
1	1.5		x	x			
2	1.52			x			
LSD	0.129						
Microp.rate NF2 Ag salt							
5	2.07	x					
1	2.5		x				
4	5.14			x			
3	5.35			x			
2	5.95				x		
LSD	0.409						
