# Supplementary material for: Influence of the Abiotic Elicitors Ag Salts of Aspartic Acid Derivatives, Self-Organized in Nanofibers with Monomeric and Dimeric Molecular Structures, on the Antioxidant Activity and Stevioside Content in Micropropagated Stevia rebaudiana Bert
Source: Plants (Basel). 2023 Oct 14;12(20):3574. doi: 10.3390/plants12203574 (PMC10610515; doi:10.3390/plants12203574)
Supplement: Supplementary file 1 [file plants-12-03574-s001.zip › Table S4. WS-,LS-, Flav., Phen., DPPH, FRAP.pdf]

WS-NF1 Ag salt					
2	50.03	x			
5	58.72		x		
4	59.103		x		
1	59.91		x		
3	60.27		x		
LSD	5.253				
WS-NF2 Ag salt					
1	59.91	x			
4	87.303		x		
5	91.81		x		
2	104.07			x	
3	115.29				x
LSD	8.507				

LS-NF1 Ag salt					
5	16.0	x			
1	16.13	x			
4	22.0		x		
2	25.73			x	
3	39.45				x
LSD	2.309				
LS-NF2 Ag salt					
4	5.63	x			
5	8.75		x		
3	11.88			x	
2	11.88			x	
1	16.13				x
LSD	1.037				

Phenols NF1 Ag salt						
5	0.6	x				
4	1.103		x			
1	2.33			x		
2	3.08				x	
3	4.05					x
LSD	0.231					
Phenols NF2 Ag salt						
1	2.33	x				
2	18.93		x			
5	21.2			x		
3	22.22			x		
4	27.68				x	
LSD	1.851					

Flavon.- NF1 Ag salt						
1	4.58	x				
5	7.25		x			
4	9.02			x		
2	10.67				x	
3	15.65					x
LSD	0.918					
Flavon.- NF2 Ag salt						
1	4.58	x				
2	6.39		x			
5	7.12		x	x		
3	7.76			x		
4	13.41				x	
LSD	0.766					

DPPH- NF1 Ag salt				
5	68.45	x		
4	68.92	x	x	
3	69.83	x	x	
1	71.28	x	x	
2	74.89		x	
LSD	6.429			
DPPH- NF2 Ag salt				
1	71.28	x		
5	90.0		x	
2	93.703		x	
4	94.303		x	
3	94.6		x	
LSD	8.115			

FRAP-NF1 Ag salt					
2	19.41	x			
5	26.0		x		
4	56.9			x	
3	57.26			x	
1	86.62				x
LSD	4.992				
FRAP-NF2 Ag salt					
4	72.05	x			
3	78.33	x			
1	86.62		x		
2	94.67			x	
5	103.03				x
LSD	7.97				
