# Supplementary material for: Influence of the Abiotic Elicitors Ag Salts of Aspartic Acid Derivatives, Self-Organized in Nanofibers with Monomeric and Dimeric Molecular Structures, on the Antioxidant Activity and Stevioside Content in Micropropagated Stevia rebaudiana Bert
Source: Plants (Basel). 2023 Oct 14;12(20):3574. doi: 10.3390/plants12203574 (PMC10610515; doi:10.3390/plants12203574)
Supplement: Supplementary file 1 [file plants-12-03574-s001.zip › Table S5. Steviosides.pdf]

| Stevioside | | | | | | |
|---|---|---|---|---|---|---|
| 5 | 2.4 | x | | | | |
| 4 | 2.94 | x | x | | | |
| 3 | 3.63 | | x | | | |
| 2 | 3.72 | | x | | | |
| 6 | 10.25 | | | x | | |
| 9 | 10.98 | | | x | x | |
| 7 | 11.0 | | | x | x | |
| 8 | 11.65 | | | | x | |
| 1 | 18.61 | | | | | x |
| LSD | 0.843 | | | | | |

| RebaudiosideA | | | | | | | | | |
|---|---|---|---|---|---|---|---|---|---|
| 4 | 0.23 | x | | | | | | | |
| 5 | 1.17 | | x | | | | | | |
| 3 | 1.93 | | | x | | | | | |
| 2 | 2.95 | | | | x | | | | |
| 9 | 3.25 | | | | x | x | | | |
| 6 | 3.58 | | | | | x | x | | |
| 8 | 3.8 | | | | | | x | x | |
| 7 | 4.12 | | | | | | | x | |
| 1 | 10.47 | | | | | | | | x |
| LSD | 0.382 | | | | | | | | |

| Sugars | | | | | | | | |
|---|---|---|---|---|---|---|---|---|
| 6 | 10.76 | x | | | | | | |
| 9 | 12.41 | x | x | | | | | |
| 1 | 15.25 | | x | | | | | |
| 8 | 19.52 | | | x | | | | |
| 7 | 20.18 | | | x | | | | |
| 3 | 34.81 | | | | x | | | |
| 5 | 39.35 | | | | | x | | |
| 4 | 42.42 | | | | | | x | |
| 2 | 64.28 | | | | | | | x |
| LSD | 2.856 | | | | | | | |
