# Supplementary material for: Influence of the Abiotic Elicitors Ag Salts of Aspartic Acid Derivatives, Self-Organized in Nanofibers with Monomeric and Dimeric Molecular Structures, on the Antioxidant Activity and Stevioside Content in Micropropagated Stevia rebaudiana Bert
Source: Plants (Basel). 2023 Oct 14;12(20):3574. doi: 10.3390/plants12203574 (PMC10610515; doi:10.3390/plants12203574)
Supplement: Supplementary file 1 [file plants-12-03574-s001.zip › Table S6. Morphol.parameters.pdf]

FWshoot NF1Ag salt					
1	0.115	x			
5	0.237		x		
2	0.311			x	
3	0.374				x
4	0.389				x
LSD	0.028				
DWshoot NF1Ag salt					
1	0.018	x			
5	0.02	x			
2	0.043		x		
4	0.066			x	
3	0.076				x
LSD	0.005				
Shoot height NF1 Ag salt					
5	2.01	x			
4	5.48		x		
1	5.97		x	x	
3	6.39			x	
2	10.14				x
LSD	0.594				
Shoot number NF1 Ag salt					
1	1.0	x			
5	1.44		x		
2	1.45		x		
3	1.99			x	
4	3.07				x
LSD	0.175				
Root init. NF1 Ag salt					
1	0.0	x			
3	5.13		x		
4	5.17		x		
5	5.26		x		
2	8.0			x	
LSD	0.489				
No internodes NF1 Ag salt					
5	2.03	x			
1	2.5		x		
2	3.9			x	
4	4.9				x

3	5.1				x
LSD	0.354				
Internodes length NF1 Ag salt					
5	1.01	x			
4	1.08	x			
3	1.25		x		
1	1.5			x	
2	2.6				x
LSD	0.145				
Microp.rate NF1 Ag salt					
1	2.5	x			
5	2.92	x			
2	5.66		x		
3	10.15			x	
4	15.04				x
LSD	0.789				
